# Global, regional, and national burden of chronic kidney disease attributable to high fasting plasma glucose from 1990 to 2019: a systematic analysis from the global burden of disease study 2019

**DOI:** 10.3389/fendo.2024.1379634

**Published:** 2024-03-27

**Authors:** Huizhi Wei, Jinhong Ren, Rui Li, Xiaoming Qi, Fan Yang, Qingshan Li

**Affiliations:** ^1^ School of Pharmaceutical Science, Medicinal Basic Research Innovation Center of Chronic Kidney Disease, Ministry of Education, Shanxi Medical University, Taiyuan, China; ^2^ Shanxi Key Laboratory of Innovative Drug for the Treatment of Serious Diseases Basing on the Chronic Inflammation, Shanxi University of Chinese Medicine, Taiyuan, China

**Keywords:** GBD 2019, mortality, disability-adjusted life year, chronic kidney disease, high fasting plasma glucose

## Abstract

**Purpose:**

Given the rising prevalence of high fasting plasma glucose (HFPG) over the past three decades, it is crucial to assess its global, national, and regional impact on chronic kidney disease (CKD). This study aims to investigate the burden of CKD attributed to HFPG and its distribution across various levels.

**Methods and materials:**

The data for this research was sourced from the Global Burden of Diseases Study 2019. To estimate the burden of CKD attributed to HFPG, we utilized DisMod-MR 2.1, a Bayesian meta-regression tool. The burden was measured using age-standardized mortality rate (ASMR) and age-standardized disability-adjusted life years (DALYs) rate. Correlation analysis was performed using the Spearman rank order correlation method. Temporal trends were analyzed by estimating the estimated annual percentage change (EAPC).

**Results:**

Globally in 2019, there were a total of 487.97 thousand deaths and 13,093.42 thousand DALYs attributed to CKD attributed to HFPG, which represent a substantial increase of 153.8% and 120%, respectively, compared to 1990. Over the period from 1990 to 2019, the burden of CKD attributable to HFPG increased across all regions, with the highest increases observed in regions with high socio-demographic index (SDI) and middle SDI. Regions with lower SDI exhibited higher ASMR and age-standardized DALYs (ASDR) compared to developed nations at the regional level. Additionally, the EAPC values, which indicate the rate of increase, were significantly higher in these regions compared to developed nations. Notably, high-income North America, belonging to the high SDI regions, experienced the greatest increase in both ASMR and ASDR over the past three decades. Furthermore, throughout the years from 1990 to 2019, males bore a greater burden of CKD attributable to HFPG.

**Conclusion:**

With an increasing population and changing dietary patterns, the burden of CKD attributed to HFPG is expected to worsen. From 1990 to 2019, males and developing regions have experienced a more significant burden. Notably, the EAPC values for both ASMR and ASDR were higher in males and regions with lower SDI (excluding high-income North America). This emphasizes the pressing requirement for effective interventions to reduce the burden of CKD attributable to HFPG.

## Introduction

Chronic kidney disease (CKD) is a condition characterized by abnormalities in kidney structure or function that persist for at least 3 months and have health implications, whose diagnostic thresholds are an estimated glomerular filtration rate of less than 60 mL/min/1.73 m^2^ and an albumin-creatinine ratio of 30 mg/g or more, as defined by the KDIGO 2012 guidelines (1), and been identified as a major global public health concern, with its prevalence and incidence increasing by 40% over the past 30 years due to global population growth and aging (2). Furthermore, recent reports indicate that there are approximately 697 million cases of CKD worldwide, contributing to a significant burden with 41.5 million disability-adjusted life years (DALYs) associated with the condition (3). Additionally, CKD poses a significant risk of accelerated cardiovascular disease and progression to end-stage renal disease (ESRD). ESRD is a grave complication associated with a poor prognosis that necessitates expensive renal replacement therapy such as dialysis or transplantation. These treatments not only adversely impact the quality of life for affected individuals but also contribute to a substantial global disease burden (4), and it is demonstrated that the overall lifetime risk of developing CKD stage 3a or higher, stage 3b or higher, stage 4 or higher, and ESRD is approximately 59.1%, 33.6%, 11.5%, and 3.6%, respectively (5). Recognizing the significant burden that CKD imposes on global health, numerous countries have taken proactive measures to raise public awareness, develop prevention and intervention strategies, and establish comprehensive care for individuals affected by CKD (6).

High fasting plasma glucose (HFPG) is a widely recognized risk factor for CKD. On one side, it can induce diabetic nephropathy; on the other side, high glucose would have a direct impact on kidney function, and it is reported that HFPG contributed to 33.59% of deaths and 30.89% of DALYs of CKD (7, 8). Due to population ageing, urbanization, industrialisation, changing dietary patterns, and the increased prevalence of obesity and low physical activity, the global mean fasting plasma glucose level is rising and HFPG is becoming more common (8), it is reported that HFPG was globally responsible for more than 6.3 million deaths and 168 million DALYs from non-communicable diseases, accounted for 11.3% of global age-standardized deaths and 6.4% of DLAYs across all causes in 2019 (3). Previous evidence has also suggested that HFPG can contribute to an increased risk of several health outcomes, such as ischemic heart disease, bladder cancer (9) and tuberculosis (10), thereby leading to a more severer healthy burden and finical cost to all countries and territories worldwide.

Given the substantial increase in the prevalence of HFPG over the past three decades (8), it is essential to assess the impact of HFPG on the burden of CKD. Understanding the extent to which CKD can be attributed to HFPG is crucial for the development of effective preventive measures for this disease in the future. Previous studies on CKD have primarily focused on describing the prevalence and attributable burden of all risk factors or high sodium intake, as a result, the burden associated specifically with HFPG on a global scale has not been adequately explored or demonstrated (6, 11, 12). The Global Burden of Diseases, Injuries, and Risk Factors Study 2019 (GBD 2019) systematically reviewed and combined risk data from 84 different risk factors. This comprehensive approach presents a unique opportunity to explore the epidemiology of CKD attributable to HFPG at the global, regional, and national levels. The aim of this research is to examine current trends in mortality and DALYs rates associated with CKD attributable to HFPG globally, regionally, and nationally. These insights can be immensely valuable for medical professionals and policymakers, facilitating informed decision-making and the development of effective management strategies.

## Materials and methods

### Data resource and disease definition

We extracted input data from the GBD 2019 database (available at http://ghdx.healthdata.org/gbd-results-tool) to estimate the burden of CKD. Clinical data sources for CKD included hospital records, emergency department records, insurance claims, surveys, and vital registration systems worldwide. The methodology for data inputting, mortality estimation, and modeling for GBD 2019 has been thoroughly demonstrated in previously published research articles, providing a comprehensive understanding of the methods utilized for this study (3, 13). Our research specifically examines the burden of CKD associated with HFPG in 204 countries and territories from 1990 to 2019. The definition of CKD used in this study aligns with the International Statistical Classification of Diseases and Related Health Problems, 10th Revision (ICD-10), and encompasses corresponding codes N18.1-18.5 and N18.9 (3). Additionally, in line with the parent GBD risk factor study, HFPG was defined as any level above the Theoretical Minimum-Risk Exposure Level (TMREL), which was 4.8 to 5.4 mmol/L (13).

### Socio-demographic index

The burden of CKD attributable to HFPG was assessed based on the level of development at the country level, as determined by the Socio-Demographic Index (SDI) (14). The SDI is a composite indicator that combines three separate indicators: lag-distributed income per capita, average educational attainment for individuals aged 15 years and older, and the total fertility rate among individuals aged younger than 25 years. Using these criteria, the 204 countries and territories were categorized into five groups based on their SDI values: low SDI (<0.45), low-middle SDI (≥0.45 and<0.61), middle SDI (≥0.61 and<0.69), high-middle SDI (≥0.69 and<0.80), and high SDI (≥0.80).

### Risk factors

In GBD 2019, risk factors were evaluated using a six-step comparative risk assessment framework. The first step involved identifying risk-outcome pairs, with only outcomes meeting the World Cancer Research Fund’s convincing or plausible evidence criteria being included (13). Next, the relative risk (RR) for each risk-outcome pair was estimated based on exposure. To obtain an accurate RR estimation, GBD 2019 conducted meta-analyses of published systematic reviews, with 81 new systematic reviews added. The third step involved examining household surveys, censuses, published studies, and governmental data to determine mean levels of risk exposure. Next, the TMREL was determined, with GBD 2019 setting it at 4.8-5.4 mmol/L. Finally, the study calculated the population attributable fraction (PAF) and attributable burden. PAF refers to the percentage of disease burden that could be reduced if exposure to a specific risk factor was limited to the TMREL, and it is calculated using the following formula: $\text{PAF= }\frac{\int_{\text{x}=1}^{\text{m}}\text{RR}(\text{x})\text{dx}-\text{RR}(\text{x})\text{TRMEL}}{\int_{x=1}^{m}\text{RR}(\text{x})\text{P}(\text{x})\text{dx}}$ . In this formula, l represents the minimum exposure level, while m represents the maximum exposure level. Finally, the study estimated the PAF and attributable burden for different combinations of risk factors. It is worth noting that the methodology for these steps has been systematically demonstrated in previous GBD research (13).

### Statistical analysis

In our latest research, we employed a robust evaluation framework by utilizing the age-standardized mortality rate (ASMR) and DALYs rate (ASDR) to assess the impact of CKD on both regional and national levels. To account for statistical uncertainty, we also calculated the corresponding 95% uncertainty intervals (95%UI) for these rates. By adjusting for standardized age structures and demographic characteristics, our study offers a comprehensive understanding of the epidemiology of CKD associated with HFPG. Previous articles have thoroughly explored and discussed the topic at hand, providing valuable insights into the field (13). The study employed age-standardized rates (ASRs) to examine trends over time by calculating the estimated annual percentage change (EAPC) in both the ASMR and ASDR from 1990 to 2019. All metrics were presented with a 95% confidence interval (95% CI) to account for statistical uncertainty. The EAPC was estimated using a linear relationship model represented by the formula: y = a + bx + e, where y represents the logarithm of ASR, x represents the calendar year, and b represents the regression coefficient. The EAPC was calculated as EAPC = 100 * (10^b-1) based on this model. To assess trends in ASMR and ASDR, the lower boundary of the 95% CI was evaluated. If the lower boundary is above zero, it indicates an upward trend, and vice versa. In order to estimate the expected values of ASMR and ASDR within each SDI unit, Gaussian process regression with a Loess smoother was used. Furthermore, Spearman’s rank order correlation was applied to evaluate the correlation between the SDI and ASMR/ASDR. Statistical significance was determined by a p-value< 0.05. All statistical analyses were performed using R software (version 4.1.0; https://cran.r-project.org).

## Results

### Global, regional and national burden of CKD attributable to HFPG from 1990 to 2019

**Figure 1:**
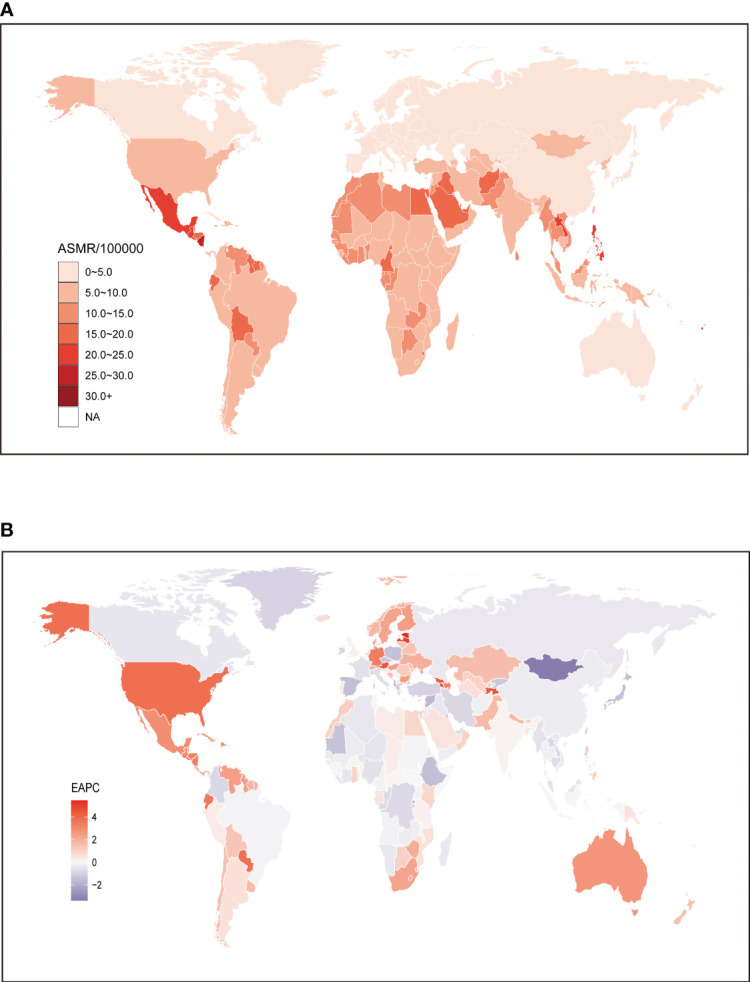
Global age standardized mortality rate of chronic kidney disease attributable to high fasting plasma glucose. **(A)** The all-cause ASMR per 100,000 associated with chronic kidney disease attributable to high fasting plasma glucose, for both sexes in 204 countries and territories in 2019. **(B)** The EAPC of ASMR of chronic kidney disease attributable to high fasting plasma glucose, for both sexes from 1990 to 2019, in 204 countries and territories. ASMR, age standardized mortality rate; EAPC, estimated annual percentage change.

At the global level, the number of deaths attributed to CKD associated with HFPG showed a significant increase from 192.26 thousand (95% UI: 157.83 to 226.61) in 1990 to 487.97 thousand (95% UI: 405.86 to 572.85) in 2019, more than tripling over this period. Similarly, the ASMR also experienced a notable change, rising from 5.1 (95% UI: 4.21 to 6.01) in 1990 to 6.14 (95% UI: 5.11 to 7.23) in 2019. Analyzing the data at a regional level, Central Latin America exhibited the highest ASMR, recording 15.92 (95% UI: 12.7 to 19.44), followed by Southeast Asia with 12.59 (95% UI: 10.59 to 14.6), and Andean Latin America with 11.33 (95% UI: 8.63 to 14.41). Conversely, Eastern Europe displayed the lowest ASMR at 0.97 (95% UI: 0.72 to 1.25) (**Table 1**). When examining individual countries, those with higher ASMR were predominantly located in Latin America, Africa, and North America. Notably, Mauritius had the highest ASMR at 38.72 (95% UI: 30.87 to 47.8), followed by Micronesia (Federated States of) at 35.21 (95% UI: 25.54 to 46.8). In 2019, countries in East Asia, Southeastern Asia, and Australasia generally experienced lower ASMR, with the exception of the Lao People’s Democratic Republic at 20.37 (95% UI: 15.64 to 26.26). Among these countries, Belarus had the lowest ASMR at 0.59 (95% UI: 0.41 to 0.84), followed by Ukraine at 0.7 (95% UI: 0.5 to 0.94), and Lithuania at 0.8 (95% UI: 0.54 to 1.11) (**Figure 1A**; **Supplementary Table S1**).

**Table 1:** Global and regional number of deaths and age-standardized morality of chronic kidney disease attributable to High fasting plasma glucose for both sexes combined in 1990 and 2019, and EAPC of ASMR from 1990 to 2019.

	Deaths number (×1000) in 1990	Deaths number (×1000) in 2019	ASMR in 1990	ASMR in 2019	EAPC 1990-2019
**Global**	192.26(157.83 to 226.61)	487.97(405.86 to 572.85)	5.1(4.21 to 6.01)	6.14(5.11 to 7.23)	0.78 (0.66 to 0.9)
Gender					
Male	99.01(79.97 to 117.53)	251.5(206.57 to 298.73)	5.95(4.84 to 7.1)	7.04(5.83 to 8.38)	0.73 (0.6 to 0.86)
Female	93.25(77.32 to 108.9)	236.47(193.35 to 280.53)	4.5(3.72 to 5.28)	5.42(4.44 to 6.43)	0.75 (0.64 to 0.86)
SDI					
High SDI	28.26(22.61 to 34.44)	85.82(67.44 to 105.56)	2.7(2.18 to 3.26)	4.06(3.22 to 4.91)	1.6 (1.42 to 1.79)
High-middle SDI	36.34(29.79 to 42.86)	72.8(59.63 to 86.7)	3.69(3.04 to 4.38)	3.68(3.02 to 4.39)	0.12 (-0.04 to 0.28)
Middle SDI	71.84(60.63 to 82.8)	191.85(159.67 to 224.36)	7.61(6.43 to 8.87)	8.37(7.02 to 9.82)	0.53 (0.45 to 0.61)
Low-middle SDI	38.61(30.73 to 47.13)	100.16(80.87 to 120.56)	6.92(5.55 to 8.45)	7.76(6.31 to 9.31)	0.4 (0.24 to 0.57)
Low SDI	17.08(13.28 to 20.8)	37(29.4 to 44.22)	8.18(6.31 to 10.25)	7.93(6.36 to 9.54)	-0.12 (-0.19 to -0.05)
Region					
Andean Latin America	1.49(1.18 to 1.79)	6.15(4.7 to 7.81)	7.72(6.09 to 9.32)	11.33(8.63 to 14.41)	1.54 (1.25 to 1.82)
Australasia	0.14(0.11 to 0.19)	0.61(0.42 to 0.88)	0.66(0.5 to 0.87)	1.11(0.76 to 1.55)	2.39 (2.06 to 2.73)
Caribbean	1.52(1.24 to 1.82)	4.12(3.21 to 5.11)	6.03(4.96 to 7.18)	7.96(6.16 to 9.87)	1.42 (1.29 to 1.55)
Central Asia	1.43(1.08 to 1.82)	3.47(2.7 to 4.26)	3.03(2.31 to 3.87)	4.97(3.92 to 6.09)	1.57 (1.16 to 1.98)
Central Europe	3.11(2.33 to 3.91)	4.58(3.35 to 6.03)	2.2(1.67 to 2.73)	2.07(1.53 to 2.71)	0.05 (-0.19 to 0.29)
Central Latin America	6.4(5.29 to 7.41)	37.05(29.51 to 45.32)	8.28(6.84 to 9.67)	15.92(12.7 to 19.44)	2.49 (2.15 to 2.83)
Central Sub-Saharan Africa	1.75(1.31 to 2.25)	3.58(2.55 to 4.69)	9.23(7.13 to 11.61)	8.03(5.71 to 10.55)	-0.58 (-0.62 to -0.53)
East Asia	41.16(34.13 to 48.81)	81.38(65.62 to 97.26)	5.11(4.28 to 5.95)	4.36(3.57 to 5.19)	-0.28 (-0.4 to -0.16)
Eastern Europe	2.24(1.65 to 2.87)	3.3(2.41 to 4.29)	0.85(0.64 to 1.11)	0.97(0.72 to 1.25)	0.19 (-0.06 to 0.44)
Eastern Sub-Saharan Africa	6.01(4.67 to 7.43)	11.1(8.84 to 13.6)	9.12(7.13 to 11.46)	8.18(6.54 to 10.07)	-0.46 (-0.53 to -0.39)
High-income Asia Pacific	9.8(8.25 to 11.28)	21.54(16.62 to 26.25)	5.42(4.55 to 6.29)	3.81(3.08 to 4.54)	-1.23 (-1.36 to -1.09)
High-income North America	7.49(5.54 to 9.85)	36.45(28.07 to 44.91)	2.07(1.53 to 2.69)	5.48(4.26 to 6.71)	3.53 (3.14 to 3.93)
North Africa and Middle East	17.35(13.6 to 21.44)	39.6(30.86 to 49.81)	11.76(9.24 to 15.22)	10.48(8.24 to 13.03)	-0.26 (-0.33 to -0.18)
Oceania	0.28(0.23 to 0.34)	0.74(0.59 to 0.91)	9.32(7.7 to 11.12)	10.54(8.49 to 12.84)	0.26 (0.06 to 0.46)
South Asia	32.72(24.19 to 42.21)	95.8(74.2 to 118.73)	6.43(4.78 to 8.21)	7.16(5.6 to 8.89)	0.29 (0.02 to 0.55)
Southeast Asia	31.49(27.2 to 36)	72.48(61.4 to 84.5)	12.34(10.63 to 14.19)	12.59(10.59 to 14.6)	0.13 (0.07 to 0.19)
Southern Latin America	2.95(2.34 to 3.58)	7.02(5.5 to 8.68)	6.7(5.33 to 8.14)	8.24(6.47 to 10.16)	0.72 (0.39 to 1.04)
Southern Sub-Saharan Africa	1.49(1.12 to 1.91)	4.59(3.59 to 5.64)	5.77(4.39 to 7.46)	9.2(7.28 to 11.37)	2.19 (1.86 to 2.52)
Tropical Latin America	4.63(3.84 to 5.42)	12.89(10.58 to 15.34)	5.54(4.57 to 6.53)	5.5(4.49 to 6.58)	0.01 (-0.08 to 0.1)
Western Europe	11.53(8.54 to 15.39)	26.5(18.54 to 36.3)	1.93(1.44 to 2.53)	2.3(1.67 to 3.11)	1.06 (0.94 to 1.19)
Western Sub-Saharan Africa	7.28(5.48 to 9.3)	15.02(11.54 to 18.74)	9.44(7.16 to 12.2)	9.14(7.05 to 11.3)	-0.17 (-0.24 to -0.1)

ASMR, age-standard morality rate; EAPC, estimated annual percentage change.

To further investigate the impact of HFPG on the burden of CKD, an analysis was conducted to examine the trends from 1990 to 2019. The findings indicated similar patterns for both sexes at the global level, with an EAPC of 0.78 (95% CI: 0.66 to 0.9). This pattern also held true when analyzing data separately for males (0.73; 95% CI: 0.6 to 0.86) and females (0.75; 95% CI: 0.64 to 0.86) (**Table 1**). From 1990 to 2019, there was a notable increase in the ASMR for CKD attributed to HFPG in most regions. The largest increase was observed in the high-income North America region (3.53; 95% CI: 3.14 to 3.93), followed by Central Latin America (2.49; 95% CI: 2.15 to 2.83). In contrast, among regions where ASMR of CKD attributable to HFPG decreased, the greatest decrease was found in high-income Asia Pacific (-1.23; 95% CI: -1.36 to -1.09). Interestingly, despite both high-income North America and high-income Asia Pacific belonging to high SDI regions and having relatively lower ASMR compared to regions with lower SDI values, the trends of CKD attributable to HFPG over the past three decades differed significantly (**Table 1**). Similarly, at the country level, the majority of countries experienced an upward trend in the ASMR for CKD attributed to HFPG from 1990 to 2019. However, there were exceptions, including Mongolia, Maldives, Cyprus, Kuwait, Solomon Islands, Albania, Rwanda, and Bosnia and Herzegovina. Among these countries, the most significant decrease was observed in Mongolia (-3.38; 95% CI: -3.85 to -2.9). On the other hand, among countries with increased ASMR over the same period, the highest increase was seen in Estonia (5.45; 95% CI: 4.85 to 6.06), closely followed by Armenia (5; 95% CI: 4.65 to 5.36) and Latvia (4.98; 95% CI: 4.42 to 5.54). These findings highlight the varying trends in the burden of CKD associated with HFPG across different countries (**Figure 1B**; **Supplementary Table S1**).

**Figure 2:**
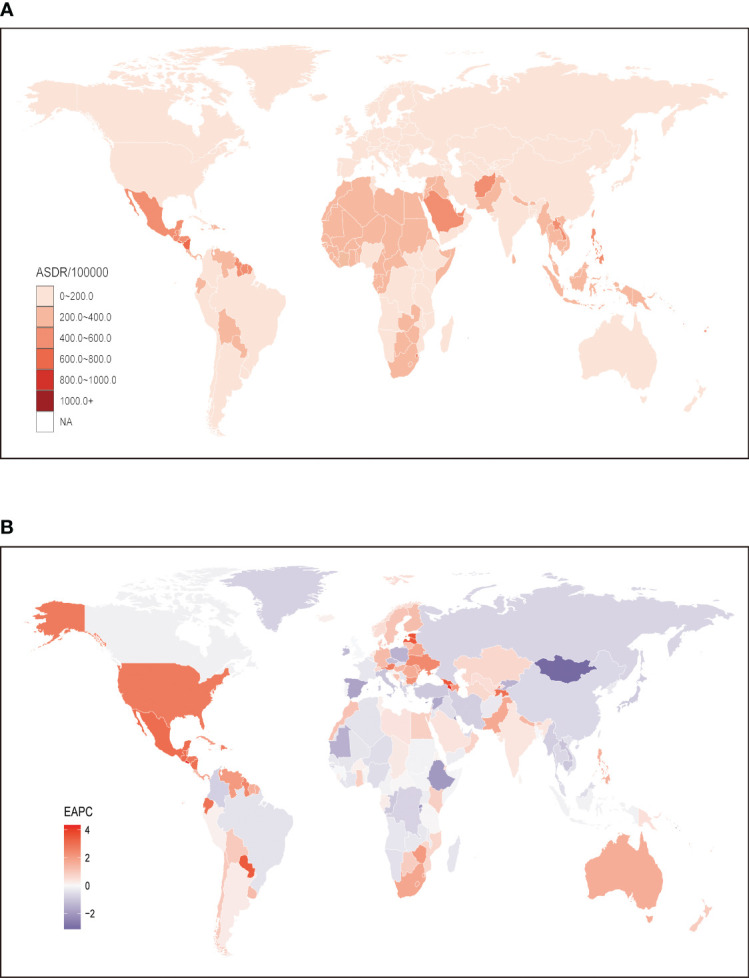
Global age standardized DALYs rate of chronic kidney disease attributable to high fasting plasma glucose. **(A)** The all-cause ASDR per 100,000 associated with chronic kidney disease attributable to high fasting plasma glucose, for both sexes in 204 countries and territories in 2019. **(B)** The EAPC of ASDR of chronic kidney disease attributable to high fasting plasma glucose, for both sexes from 1990 to 2019, in 204 countries and territories. DALYs, disease adjusted life year. ASDR, age standardized DALYs rate; EAPC, estimated annual percentage change.

Globally, the burden of CKD attributed to HFPG has increased significantly over the past three decades. The number of DALYs attributed to CKD caused by HFPG has risen from 5,950.23 thousand (95% UI: 5,013.38 to 6,909.91) in 1990 to 13,093.42 thousand (95% UI: 11,103.9 to 15,211.6) in 2019, representing a staggering 120% increase. Moreover, there has been a more noticeable increase in ASDR, with the global ASDR of CKD attributable to HFPG increasing from 141.19 (95% UI: 119 to 164.03) in 1990 to 159.03 (95% UI: 135.36 to 184.35) in 2019. At the regional level, it is worth noting that regions with lower SDI face a more significant burden of ASDR for CKD. Among these regions, Central Latin America exhibited the highest ASDR globally at 414.82 (95% UI: 337.62 to 497.97), followed by other developing regions such as Southeast Asia (338.84; 95% UI: 291.46 to 389.67), Oceania (304.1; 95% UI: 247.36 to 366.31), and Andean Latin America (248.58; 95% UI: 191.4 to 312.73). Conversely, regions with higher SDI generally displayed lower ASDRs when compared to the aforementioned low or low-middle SDI regions. The lowest ASDR was observed in Australasia at 31.21 (95% UI: 23.93 to 39.87), followed by Eastern Europe (36.44; 95% UI: 28.55 to 45.46) and Western Europe (44.67; 2.44; 95% UI: 35.4 to 55.84). However, it is worth noting that high-income North America, a region primarily composed of developed countries, is facing a remarkably high ASDR for CKD at 130.92 (95% UI: 106.59 to 155) (**Table 2**). When analyzing trends at the country level, it is evident that developed countries generally face a lighter burden of CKD attributable to HFPG. Nonetheless, it is concerning to note that Mauritius (1023.49; 95% UI: 813.85 to 1268.02) is facing the highest ASDR of CKD attributable to HFPG, followed by Micronesia (Federated States of) (972.55; 95% UI: 659.5 to 1321.21), Palau (867; 95% UI: 659.79 to 1107.73), and Nauru (837.89; 95% UI: 591.41 to 1093.74). On the other hand, Iceland (22.03; 95% UI: 16.97 to 28.79) has the lowest ASDR, followed by Belarus (23.62; 95% UI: 17.91 to 30.97), Ukraine (26.69; 95% UI: 20.46 to 34.82), and the United Kingdom (28.17; 95% UI: 21.98 to 35.32), which are characterized by developed finance and a health dirt pattern (**Figure 2A**; **Supplementary Table S2**).

**Table 2:** Global and regional number of DALYs and age-standardized DALYs rate of chronic kidney disease attributable to high fasting plasma glucose for both sexes combined in 1990 and 2019, and EAPC of ASMR from 1990 to 2019.

	DALYs number (×1000) in 1990	DALYs number (×1000) in 2019	ASDR in 1990	ASDR in 2019	EAPC 1990-2019
**Global**	5950.23(5013.38 to 6909.91)	13093.42(11103.9 to 15211.6)	141.19(119 to 164.03)	159.03(135.36 to 184.35)	0.53 (0.43 to 0.64)
Gender					
Male	3119.49(2595.34 to 3658.27)	7001.9(5849.96 to 8220.11)	157.21(131.04 to 183.59)	178.96(149.77 to 209.81)	0.61 (0.48 to 0.73)
Female	2830.73(2381.68 to 3278.7)	6091.52(5158.86 to 7078.91)	128.13(107.91 to 148.47)	141.09(119.9 to 164.21)	0.41 (0.32 to 0.51)
SDI					
High SDI	741.17(619.7 to 866.03)	1750.65(1455.84 to 2056.92)	72.64(61.05 to 84.81)	96.84(81.26 to 113.19)	1.14 (0.98 to 1.31)
High-middle SDI	1106.58(936.41 to 1290.49)	1911.43(1613.17 to 2240.41)	101.65(86.35 to 118.36)	96.45(81.9 to 112.38)	-0.07 (-0.22 to 0.07)
Middle SDI	2351.02(2012.64 to 2710.4)	5403.12(4585.11 to 6235.11)	200.84(172.19 to 229.66)	212.04(180.62 to 244.46)	0.38 (0.3 to 0.46)
Low-middle SDI	1243.88(1015.58 to 1495.17)	2929.27(2407.26 to 3480.13)	184.69(149.23 to 222.17)	202.73(166.81 to 241.9)	0.37 (0.22 to 0.52)
Low SDI	503.4(397.35 to 615.05)	1089.18(873.47 to 1306.43)	195.84(155.74 to 237.09)	191.39(154.07 to 228.82)	-0.08 (-0.15 to 0)
Region					
Andean Latin America	39.82(32.03 to 47.24)	140.32(107.53 to 177.09)	182.17(146.58 to 217.23)	248.58(191.4 to 312.73)	1.24 (0.97 to 1.51)
Australasia	5.09(4.11 to 6.29)	15.01(11.45 to 19.2)	22.2(17.99 to 27.44)	31.21(23.93 to 39.87)	1.46 (1.25 to 1.68)
Caribbean	44.34(36.94 to 52.65)	113.6(89.48 to 140.04)	163.81(136.48 to 194.17)	220.41(173.72 to 271.33)	1.4 (1.3 to 1.5)
Central Asia	49.66(38.92 to 62.64)	115.55(92.35 to 140.33)	96.87(76.04 to 121.91)	141.03(113 to 169.66)	1.01 (0.63 to 1.38)
Central Europe	90.69(70.39 to 110.99)	117.4(90.44 to 146.91)	62(48.82 to 75.84)	57.49(44.81 to 71.7)	-0.05 (-0.21 to 0.1)
Central Latin America	194.1(164.02 to 224.07)	1004.07(809.97 to 1210.43)	212.24(177.85 to 244.08)	414.82(337.62 to 497.97)	2.51 (2.18 to 2.85)
Central Sub-Saharan Africa	51.44(38.8 to 65.95)	104.74(75.41 to 136.54)	208.36(159.01 to 262.1)	178.87(130.03 to 232.4)	-0.62 (-0.67 to -0.57)
East Asia	1438.92(1200.03 to 1696.23)	2288.85(1898.9 to 2702.08)	145.9(122.62 to 170.66)	112.91(94.22 to 132.1)	-0.6 (-0.72 to -0.48)
Eastern Europe	90.76(71.31 to 114.15)	116.48(90.07 to 145.93)	33.68(26.76 to 42.32)	36.44(28.55 to 45.46)	-0.07 (-0.29 to 0.16)
Eastern Sub-Saharan Africa	171.38(135.2 to 209.79)	304.43(246.39 to 367.13)	209.73(165.99 to 256)	175.95(142.05 to 212.29)	-0.71 (-0.79 to -0.63)
High-income Asia Pacific	242.79(210.93 to 273.63)	385(325.71 to 441.78)	122.62(106.71 to 138.29)	88.53(75.89 to 101.79)	-0.96 (-1.14 to -0.77)
High-income North America	219.62(173.81 to 268.32)	793.21(642.56 to 943.1)	63.96(50.34 to 78.46)	130.92(106.59 to 155)	2.6 (2.28 to 2.91)
North Africa and Middle East	461.02(369.79 to 550.37)	1060.22(849.12 to 1303.9)	261.46(209.72 to 314.95)	239.95(192.37 to 295.27)	-0.19 (-0.23 to -0.14)
Oceania	10.79(8.96 to 12.98)	27.32(22.02 to 33.4)	276.62(229.36 to 327.11)	304.1(247.36 to 366.31)	0.16 (-0.04 to 0.37)
South Asia	1039.59(790.27 to 1312.31)	2823.65(2203.58 to 3483.95)	166.89(127.95 to 209.84)	189.85(149.21 to 234.41)	0.5 (0.27 to 0.74)
Southeast Asia	1081.28(930.63 to 1234.26)	2225.05(1909.79 to 2564.91)	343.25(297.91 to 390.87)	338.84(291.46 to 389.67)	0.02 (-0.04 to 0.07)
Southern Latin America	71.65(57.54 to 84.64)	142.92(116.13 to 171.11)	155.19(125.54 to 182.95)	172.94(140.99 to 206.88)	0.36 (0.09 to 0.63)
Southern Sub-Saharan Africa	45.14(34.52 to 57.95)	125.42(98.35 to 156.37)	147.57(114.37 to 188.98)	212.28(168.05 to 262.98)	1.81 (1.49 to 2.12)
Tropical Latin America	144.23(119.5 to 168.36)	334.34(276.27 to 395.81)	144.07(120.63 to 167.53)	136.65(113.35 to 161.12)	-0.31 (-0.42 to -0.2)
Western Europe	259.63(202.29 to 322.21)	426.71(335.53 to 538.91)	45.19(35.67 to 55.87)	44.67(35.4 to 55.84)	0.17 (0.11 to 0.23)
Western Sub-Saharan Africa	198.3(150.67 to 255.29)	429.13(327.14 to 537.16)	211.16(162.03 to 267.06)	204.38(158.09 to 252.28)	-0.14 (-0.21 to -0.07)

ASDR, age-standard DALYs rate; DALYs, disability-adjusted life years; EAPC, estimated annual percentage change.

The analysis of the EAPC of the ASDR for CKD attributed to HFPG from 1990 to 2019 consistently revealed increasing trends. Globally, both males and females exhibited a continuous increase during this period, with an overall EAPC of 0.53 (95% CI: 0.43 to 0.64). Notably, males experienced a faster increase in the ASDR of CKD attributed to HFPG compared to females, with an EAPC of 0.61 (95% CI: 0.48 to 0.73) (**Table 2**). At the regional level, the EAPC values varied across regions, and no significant association between EAPC and SDI was observed. The region with the greatest increase was high-income North America (2.6; 95% CI: 2.28 to 2.91), followed by Central Latin America (2.51; 95% CI: 2.18 to 2.85) and Southern Sub-Saharan Africa (1.81; 95% CI: 1.49 to 2.12). In contrast, high-income Asia Pacific (-0.96; 95% CI: -1.14 to -0.77), a region with a high SDI, exhibited the largest decrease in ASDR over the same period (**Table 2**). At the national level, a decreasing trend in ASDR of CKD attributed to HFPG was observed in 81 countries, primarily located in regions with higher SDI, such as Eastern Europe. Among these countries, Mongolia had the lowest EAPC value (-3.12; 95% CI: -3.55 to -2.68), followed by Maldives (-2.75; 95% CI: -3.06 to -2.44) and Cyprus (-2.18; 95% CI: -2.28 to -2.07). On the other hand, El Salvador showed the highest increase in ASDR, with an EAPC value of 4.34 (95% CI: 3.63 to 5.04), followed by Armenia (4.24; 95% CI: 3.9 to 4.58), Estonia (3.62; 95% CI: 3.13 to 4.12), and Lesotho (3.58; 95% CI: 3.22 to 3.94) (**Figure 2B**; **Supplementary Table S2**).

### Correlation between the burden of CKD attributable to HFPG with SDI

**Figure 3:**
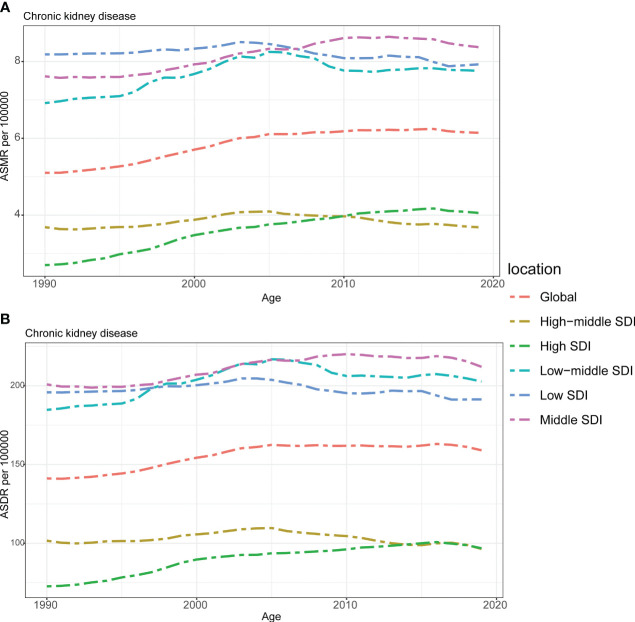
The burden of chronic kidney disease attributable to high fasting plasma glucose by SDI. **(A)** The ASMR and **(B)** ASDR of chronic kidney disease attributable to high fasting plasma glucose in different SDI regions from 1990 to 2019. Results are showed for both sexes worldwide. ASMR, age standardized mortality rate; DALYs, disease adjusted life year. ASDR, age standardized DALYs rate. SDI, sociodemographic index.

As depicted in **Figure 3**, the ASMR and ASDR of CKD attributed to HFPG exhibited a consistent upward trend from 1990 to 2019, both globally and across most regions in the GBD study. It is noteworthy that regions with lower SDI values, such as middle SDI, low SDI, and low-middle SDI regions, faced a greater burden of ASMR and ASDR compared to the global average. Conversely, regions with higher SDI values experienced a relatively lower mortality burden, with ASMR and ASDR rates below the global average. However, it is important to highlight that while regions with higher SDI, such as high SDI and high-middle SDI regions, had a lighter burden compared to regions with lower SDI values, the rate of increase in both ASMR and ASDR was consistently higher in high SDI regions. In fact, the rate of increase in these higher SDI regions surpassed that in regions with lower SDI over the years. Meanwhile, the rate of increase in high-middle SDI, low-middle SDI and low SDI regions showed a gradual decline during this period (**Figure 3**).

**Figure 4:**
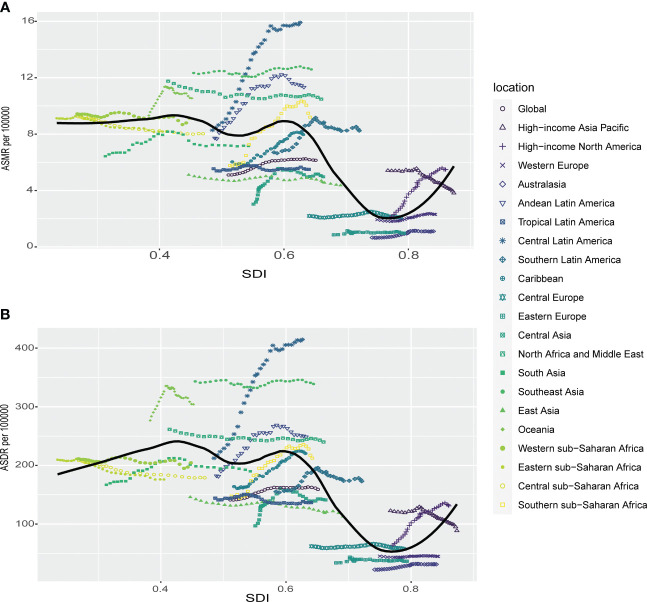
Correlations of ASMR as well as ASDR of chronic kidney disease attributable to high fasting plasma glucose and SDI at the regional level. The ASMR **(A)** and ASDR **(B)** of chronic kidney disease attributable to high fasting plasma glucose and SDI at the regional level in 21 regions from 1990 to 2019. ASMR, age standardized mortality rate; DALYs, disease adjusted life year. ASDR, age standardized DALYs rate.

Correlation analysis was conducted to explore the relationship between SDI and regions as well as nations in relation to ASMR and ASDR of CKD attributed to HFPG. Our findings revealed overall negative associations, indicating that higher SDI levels were generally associated with lower ASMR and ASDR rates (**Figures 4**, **S1**). At the regional level, high-income Asia Pacific, high-income North America, and South Asia exhibited higher ASMR than anticipated based on their SDI values during the observed period. Conversely, Eastern Europe, Australasia, Western Europe, and East Asia experienced lower ASMR rates than expected given their SDI levels (**Figure 4A**). The same phenomenon was also seen in the results of ASDR for these regions (**Figure 4B**). The findings of our research, comparing the observed ASMR and ASDR with the expected levels based on SDI values at the national level, were consistent with those at the regional level. While the Mauritius had the highest ASMR and ASDR rates in 2019 as well as largest disparity in both ASMR and ASDR for CKD attributed to HFPG according to our study (**Supplementary Figure S1**).

### Age and sex patterns

**Figure 5:**
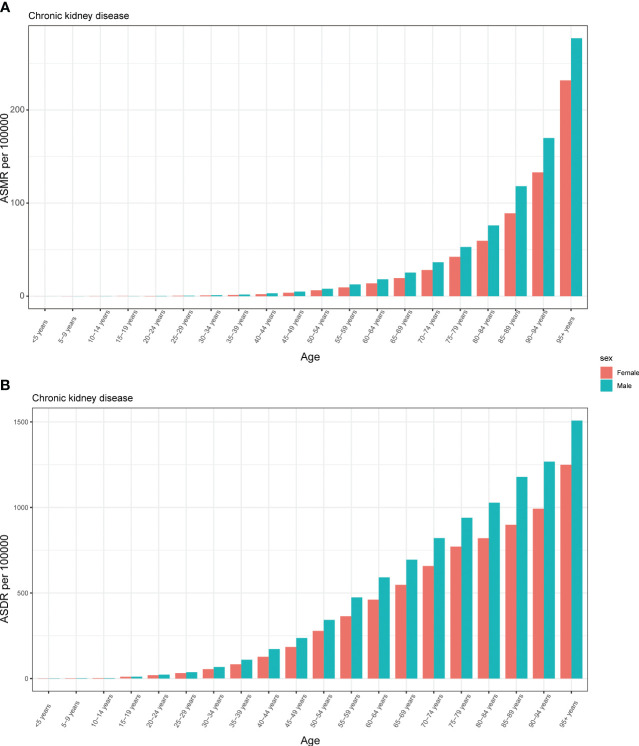
The burden of chronic kidney disease attributable to high fasting plasma glucose by age and sex. The all-cause ASMR **(A)** and ASDR **(B)** of chronic kidney disease attributable to high fasting plasma glucose worldwide in different age groups. ASMR, age standardized mortality rate; DALYs, disease adjusted life year. ASDR, age standardized DALYs rate.

In 2019, it was found that males had higher ASMR per 100,000 across all age groups, except for individuals aged less than 10 years. Additionally, this disparity in ASMR between males and females increased and then decreased with age, reaching its peak in the 35-39 age group (**Figure 5A**). Our research also indicated that males were predominantly affected by ASDR of CKD attributed to HFPG. However, the largest difference between males and females was observed among individuals aged 45-49 years, and this difference decreased with increasing age. It is worth noting that there was another peak in ASDR among those aged 85 to 89 years, followed by a sharp decline among individuals aged 90-95 and 95+ years (**Figure 5B**).

**Figure 6:**
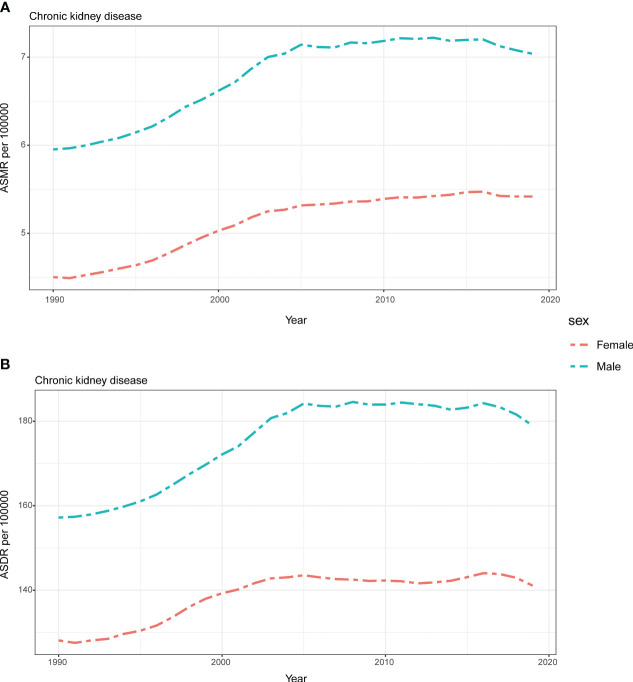
Global attributable burden of chronic kidney disease attributable to high fasting plasma glucose by sex. The age-standard ASMR **(A)** and ASDR **(B)** of chronic kidney disease attributable to high fasting plasma glucose by sex from 1990 to 2019. ASMR, age standardized mortality rate; DALYs, disease adjusted life year. ASDR, age standardized DALYs rate.

From 1990 to 2019, males consistently experienced a higher burden of CKD attributed to HFPG, as indicated by both ASMR and ASDR. Moreover, these disparities gradually increased over the years (**Figure 6**). On the other hand, the ASMR for male and females showed a slow decrease from 2015 to 2019 while the decrease in males were more apparent (**Figure 6A**). A similar pattern was observed in the ASDR for males and females, with the rates stabilizing after 2005 and fluctuating for the next 15 years (**Figure 6B**). In contrast, both ASMR and ASDR for males and females consistently rose over the thirty-year period, although there was a noticeable decrease from 2015 (**Figure 6**).

Although there was an overall increasing trend observed during the period, the ratio of male to female mortality and DALYs rates exhibited a significant difference (**Supplementary Figure S2**). The ratio of male to female DALYs rate remained consistently above zero and steadily increased at a relatively stable pace before 45 years old before gradually decreasing (**Supplementary Figure S2B**). Conversely, the growth of the ratio of male to female mortality was less than zero among those aged less than 5 years and 5-9 years, then increased at a steady speed before 19 years before eventually decreasing (**Supplementary Figure S2A**).

## Discussion

In 2019, the global number of individuals affected by all stages of CKD reached almost 700 million, surpassing the number of people with conditions such as diabetes, osteoarthritis, chronic obstructive pulmonary disease, asthma, or depressive disorders (3), and according to GBD 2019, CKD was ranked as the 12th leading cause of death out of 133 conditions by GBD (15). In this study, we conducted a comprehensive analysis to examine the burden of CKD attributed to HFPG and its associations with SDI, age, and year. Our findings revealed that on a global scale, the ASMR and ASDR of CKD attributed to HFPG remained substantial and exhibited significant increases from 1990 to 2019, particularly among males. When analyzing the burden at the regional level, regions with higher SDI values generally experienced a lower burden of CKD attributed to HFPG. For instance, Australasia, Eastern Europe, and Central Europe displayed lower rates of increase, as indicated by the EAPC value. However, it is worth noting that the rate of increase varied across regions, and no clear association between EAPC and SDI was found. However, high-income North America, primarily composed of developed countries, stands out as a region with the highest increasing speed for the burden of CKD attributed to HFPG. In fact, this burden has increased significantly over the past three decades at a rapid pace. The same trend was observed at the national level within the region. Additionally, when examining the correlation between ASMR and ASDR with SDI, a negative correlation between SDI value and both metrics was revealed. These findings suggest that regions with high SDI and high-middle SDI regions tend to have a lower burden of CKD attributed to HFPG, which is consistent with the results of our study. Furthermore, our analysis revealed that females generally exhibited lower ASMR and ASDR compared to males. The gender gap in ASDR reached its peak in the 45-49 age group, while for ASMR, it occurred in the 35-39 age group.

The rapid population growth observed in almost all regions and nations, coupled with ongoing urbanization and industrialization, has collectively contributed to the increasingly serious burden of CKD, which is reflected in the rising number of deaths and DALYs cases, as well as the increasing ASMR and ASDR. Despite the progress made in preventing mortality from several important non-communicable diseases, we have not observed the same level of success for CKD. Specifically, between 1990 and 2017, the global ASMR declined by 30.4% for cardiovascular disease, 14.9% for cancer, and 41.3% for COPD. However, during the same period, there was only a meager decrease in the ASMR for CKD (2.8%; 95% uncertainty interval: 1.5% to 6.3%) (16). Interestingly, our findings reveal an intriguing pattern where regions and countries with higher SDI values exhibit a lower burden of CKD attributed to HFPG, which underscores the significance of ensuring access to renal replacement therapy, encompassing both the initiation of treatment and the maintenance of dialysis. Additionally, public health policies play a crucial role in curbing the incidence rate of ESKD through initiatives like educating healthcare professionals, implementing early detection programs for kidney disease, promoting nephroprotective treatments, and effectively addressing CKD risk factors such as elevated systolic blood pressure and glucose levels (17). This action is particularly critical in regions with low SDI, where primary healthcare systems are primarily geared towards child and maternal health, and may be ill-equipped to effectively prevent and manage chronic diseases (18). Nevertheless, it is worth noting that some high-income countries may face resource constraints when it comes to prioritizing the prevention of CKD and its associated risk factors, including diabetes, hypertension, and obesity. This could be attributed to larger budget allocations towards healthcare services and a potential lack of awareness regarding the risk factors associated with CKD (19, 20).

In this research, it was observed that Central Latin America maintain both highest ASMR and ASDR of CKD attributed to HFPG, which align with a previously published article that aimed to determine the prevalence and burden of CKD across all causes (6). This phenomenon can potentially be attributed to the unfortunate reality of limited public health funding and a shortage of healthcare workers, which has greatly exacerbated the burden of the disease (21). Furthermore, in Central Latin America, the unaffordable costs and underutilization of cost-effective dialysis therapies, such as peritoneal dialysis, have created challenges in ensuring sufficient access to dialysis for individuals. This issue is not limited to this region alone but also extends to developed regions like Europe (21–23). It is noteworthy that starting from 1990, the ASDR of middle SDI regions has exceeded that of both low-middle SDI and low SDI regions. Similarly, from 2005, the ASMR of middle SDI regions has surpassed that of low-middle SDI and low SDI regions. One potential explanation for this trend is the difficulty in conducting comprehensive CKD examinations and the underreporting of CKD data in regions with limited medical institutions and advanced laboratory diagnostic services. This highlights the need to recognize that the association between the burden of CKD attributed to HFPG and SDI should not be regarded as simplistic or linear.

Our research findings indicate that both the ASMR and ASDR for CKD attributed to HFPG increased with age for both males and females, suggesting that the elderly population is burdened with a higher prevalence of CKD compared to younger individuals, which aligns with the findings of a previous study (6). The elderly is more susceptible to CKD due to the natural aging process and the presence of intrinsic renal diseases compared to younger individuals (24), furthermore, the increasing life expectancy and improved awareness of early testing have resulted in a growing geriatric population, which has led to a rise in the prevalence of CKD and other chronic diseases (6, 25). From 1990 to 2019, males consistently experienced a higher burden of CKD attributed to HFPG compared to females across all three decades which might be influenced by factors such as androgen levels, nitric oxide metabolism, and excessive oxidative stress (26). Given the notable differences in age, gender, and associated ASMR and ASDR of CKD, it is necessary to develop targeted health education and management strategies instead of relying on homogeneous guidance. Such tailored approaches are crucial in addressing and alleviating the challenges posed by CKD.

Consistent with the findings of our research on CKD attributed to HFPG, it is worth noting that in 2019, the global prevalence and attributable burden of HFPG were higher in low-middle SDI, low SDI, and middle SDI regions compared to high SDI and high-middle SDI regions (8). Low health literacy, inadequate medical resources, and limited preventive measures may contribute to a greater disease burden attributed to HPFG in regions with lower SDI. Conversely, residents in higher socioeconomic regions are more likely to possess higher health literacy, better access to medical treatment, maintain a healthy diet, and engage in regular exercise, all of which can help reduce the burden of CKD attributable to HFPG. It is worth noting that despite being mostly comprised of developed countries, high-income North America, a high SDI region, has experienced the greatest increase in both ASMR and ASDR over the past thirty years. This trend aligns with the prevalence of HFPG in the region. The high prevalence and substantial increase of diabetes among adults in the United States may have contributed significantly to the heavy burden of both HFPG and CKD attributed to HFPG (27).

As a primary cause of CKD, it is crucial to continuously improve the prevention, diagnosis, and treatment of HFPG on a global scale. Governments should promote a healthy lifestyle for their citizens, including advocating for a balanced diet with fewer high-fat and high-sugar foods, encouraging regular exercise, discouraging sedentary activities, and promoting smoking cessation and reduced alcohol consumption. Additionally, healthcare infrastructure and the workforce should be strengthened worldwide to ensure adequate healthcare for patients with high blood pressure and high blood glucose and to reduce the risk of developing diabetic nephropathy. Moreover, urinary tract infections (UTIs), which are considered a major risk factor for CKD in childhood, although the etiology is not yet fully understood (28), have been shown to have a high prevalence worldwide, particularly among females (29). It is reported that approximately 40–50% of women will experience at least one episode of UTI during their lifetime (29). Additionally, Micle et al. (30) demonstrated that the prevalence of UTIs was significantly higher in the third trimester of pregnancy, potentially leading to a moderate degree of neonatal impairment and an increased risk of developing CKD in these infants. Given this situation, there is an urgent need to pay more attention to reducing the prevalence of UTIs.

To the best of our knowledge, this was the most updated study to represent the most up-to-date examination of the global epidemiology of CKD attributed to HFPG, which encompasses data from 204 countries, including some that had not been previously included in similar research. It is important to highlight the pressing need for essential measures to alleviate the burden of CKD attributed to HFPG. This urgency arises from both the acute burden it presents and the potential damage of hyperglycemia on mitochondrial respiration in renal mesangial and tubular cells in humans. This study also had some limitations which were common for all GBD estimates (3, 13). First and foremost, it is important to acknowledge that the availability and completeness of data sources were not comprehensive. Moreover, in some developing countries, the scarcity of well-established medical resources contributed to the limited availability of high-quality primary data. However, the GBD project is required to generate final estimates using statistical methods and predictive covariate values. Lastly, it should be noted that GBD 2019 did not incorporate the results of some current national food surveys, such as the Family Budget Survey and others, which could have provided additional valuable insights.

## Conclusion

Over the course of the past three decades, there has been a significant increase in the burden of CKD attributable to HFPG worldwide, particularly in terms of ASDR, which has been closely aligned with the rising prevalence of HFPG. When examining regional and national levels, it is evident that high-income regions and developed countries have generally experienced a lower burden of CKD attributable to HFPG. These areas have also exhibited lower EAPC values in incidence rates, such as high-income Asia Pacific, Central Europe, and East Europe. However, it is worth noting that high‐income North America has witnessed the greatest increase in both ASMR and ASDR over the past thirty years. Moreover, throughout this thirty-year period, males consistently demonstrated higher values in both ASMR and ASDR of CKD attributable to HFPG when compared to females. Given these findings, it is crucial to prioritize additional education on healthy lifestyles and diets, particularly for males in all countries, aiming to reduce the burden of CKD attributable to HFPG and mitigate its growth, especially in regions and countries with lower SDI values.

## Data availability statement

The datasets presented in this study can be found in online repositories. The names of the repository/repositories and accession number(s) can be found in the article/**Supplementary Material**.

## Ethics statement

Ethical approval was not required for the study involving humans in accordance with the local legislation and institutional requirements. Written informed consent to participate in this study was not required from the participants or the participants’ legal guardians/next of kin in accordance with the national legislation and the institutional requirements.

## Author contributions

HW: Methodology, Writing – original draft, Writing – review & editing, Data curation, Project administration, Software, Visualization. JR: Visualization, Writing – review & editing, Supervision, Validation. RL: Software, Writing – review & editing. XQ: Writing – review & editing. FY: Project administration, Validation, Visualization, Writing – review & editing. QL: Funding acquisition, Methodology, Supervision, Validation, Writing – original draft, Writing – review & editing.
