# Supplementary material for: Global, regional, and national burden of chronic kidney disease attributable to high fasting plasma glucose from 1990 to 2019: a systematic analysis from the global burden of disease study 2019
Source: Front Endocrinol (Lausanne). 2024 Mar 27;15:1379634. doi: 10.3389/fendo.2024.1379634 (PMC11004380; doi:10.3389/fendo.2024.1379634)
Supplement: Supplementary file 1 [file DataSheet_1.docx]

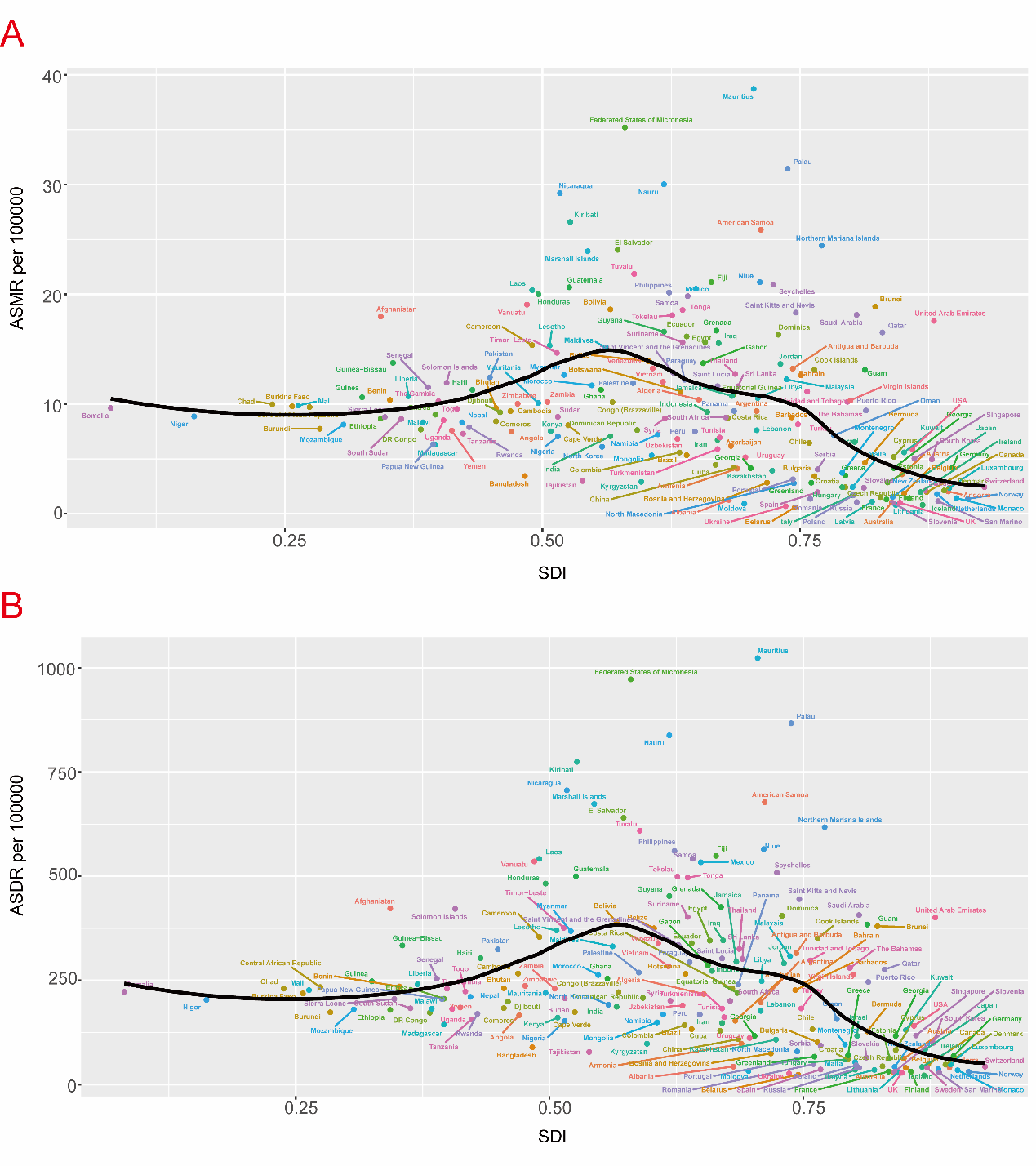


**Figure S1**. Correlations of ASMR as well as ASDR and SDI at the national level. The ASMR (A) as well as ASDR (B) of chronic kidney disease attributable to high fasting plasma glucose and SDI at the regional level in 204 countries and territories from 1990 to 2019.

ASMR, age standardized mortality rate; DALYs, disease adjusted life year. ASDR, age standardized DALYs rate; SDI, ﻿sociodemographic index.
