# Supplementary material for: Global, regional, and national burden of chronic kidney disease attributable to high fasting plasma glucose from 1990 to 2019: a systematic analysis from the global burden of disease study 2019
Source: Front Endocrinol (Lausanne). 2024 Mar 27;15:1379634. doi: 10.3389/fendo.2024.1379634 (PMC11004380; doi:10.3389/fendo.2024.1379634)
Supplement: Supplementary file 2 [file DataSheet_2.docx]

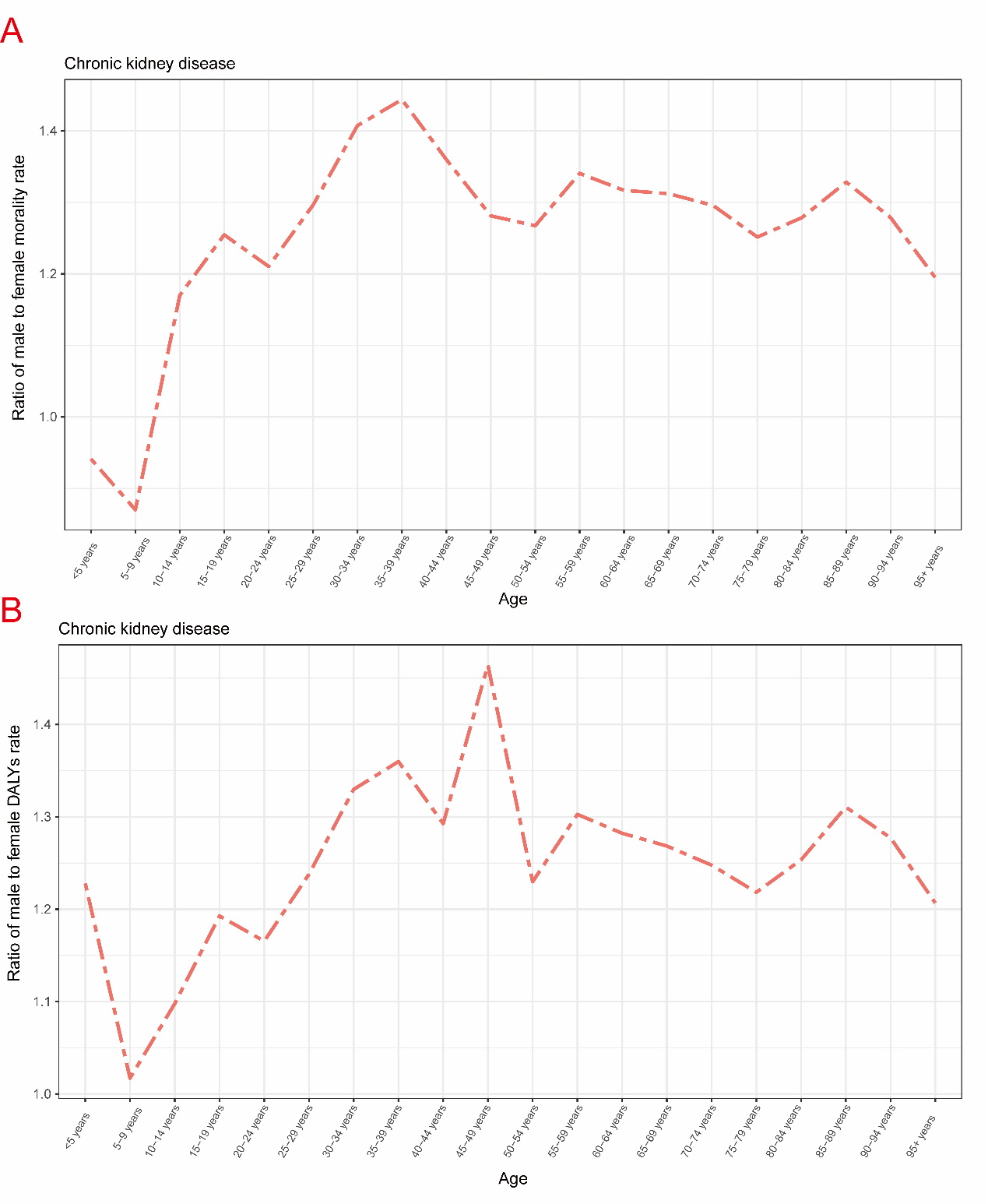


**Figure S2.** ﻿ Sex disparity in the global exposure and attributable burden of chronic kidney disease attributable to high fasting plasma glucose in different years. Ratio of male to female ASMR (A) and ASDR (B) of chronic kidney disease attributable to high fasting plasma glucose from 1990 to 2019.

ASMR, age standardized mortality rate; DALYs, disease adjusted life year. ASDR, age standardized DALYs rate.
