# Supplementary material for: Global, regional, and national burden of chronic kidney disease attributable to high fasting plasma glucose from 1990 to 2019: a systematic analysis from the global burden of disease study 2019
Source: Front Endocrinol (Lausanne). 2024 Mar 27;15:1379634. doi: 10.3389/fendo.2024.1379634 (PMC11004380; doi:10.3389/fendo.2024.1379634)
Supplement: Supplementary file 3 [file Table_1.docx]

Table S1. Death number and age-standardized morality of chronic kidney disease attributable to high fasting plasma glucose for both sexes combined in 1990 and 2019, and EAPC of ASMR from 1990 to 2019 in 204 countries and territories

| Location | Death number in 1990 | Death number in 2019 | ASMR in 1990 | ASMR in 2019 | EAPC 1990-2019 |
| --- | --- | --- | --- | --- | --- |
| Afghanistan | 1296.09(899.25 to 1907.04) | 1963.18(1386.11 to 2830.52) | 19.87(13.82 to 30.24) | 17.97(12.53 to 27.65) | -0.23 (-0.29 to -0.18) |
| Albania | 34.06(23.51 to 45.66) | 53.71(32.89 to 79.74) | 1.73(1.2 to 2.35) | 1.27(0.79 to 1.86) | -1.76 (-2.15 to -1.37) |
| Algeria | 1172.48(818.42 to 1759.68) | 2838.97(2007.47 to 3944.01) | 12.61(9.09 to 18.49) | 10.4(7.35 to 14.26) | -0.44 (-0.64 to -0.25) |
| American Samoa | 3.73(3.01 to 4.5) | 11.57(9.25 to 14.01) | 17.56(14.34 to 21.33) | 25.89(20.99 to 31.11) | 1.42 (1.28 to 1.56) |
| Andorra | 0.94(0.6 to 1.44) | 3.23(2.08 to 4.81) | 2.33(1.55 to 3.55) | 2.04(1.33 to 3.01) | -0.4 (-0.6 to -0.19) |
| Angola | 259.3(173.41 to 349.77) | 685.79(443.57 to 932.04) | 7.68(5.18 to 10.2) | 7.5(4.93 to 10.15) | -0.15 (-0.19 to -0.1) |
| Antigua and Barbuda | 4.91(4 to 5.89) | 12.5(9.7 to 15.44) | 9(7.37 to 10.83) | 13.23(10.36 to 16.36) | 1.65 (1.43 to 1.86) |
| Argentina | 2392.61(1869.98 to 2923.43) | 5168.77(4031.52 to 6423.71) | 7.74(6.07 to 9.43) | 9.37(7.33 to 11.62) | 0.57 (0.25 to 0.89) |
| Armenia | 22.6(17.66 to 28.1) | 166.12(125.33 to 211.92) | 1(0.78 to 1.26) | 4.12(3.13 to 5.26) | 5 (4.65 to 5.36) |
| Australia | 101.77(78.36 to 134.08) | 484.14(324.4 to 705.14) | 0.58(0.43 to 0.78) | 1.02(0.69 to 1.47) | 2.72 (2.38 to 3.06) |
| Austria | 172.67(125.46 to 235.81) | 800.43(559.78 to 1087.78) | 1.4(1.03 to 1.87) | 3.59(2.57 to 4.87) | 4.43 (3.82 to 5.05) |
| Azerbaijan | 149.49(113.4 to 187.29) | 491.54(371.71 to 626.8) | 2.96(2.27 to 3.74) | 6.18(4.68 to 7.81) | 3.25 (2.76 to 3.75) |
| Bahamas | 12.68(10.27 to 15.04) | 38.13(29.1 to 49.03) | 8.33(6.73 to 9.97) | 10.2(7.88 to 12.85) | 1.07 (0.92 to 1.23) |
| Bahrain | 16.96(13.04 to 21.77) | 70.67(52.46 to 91.49) | 13.97(10.82 to 17.97) | 12.62(9.53 to 16.34) | -0.17 (-0.73 to 0.38) |
| Bangladesh | 1730.03(1230.71 to 2280.8) | 4093.64(2906.7 to 5446.78) | 4.03(2.88 to 5.33) | 3.44(2.5 to 4.62) | -0.33 (-0.66 to 0) |
| Barbados | 18.55(15.19 to 22.06) | 42.59(32.78 to 53.36) | 6.31(5.21 to 7.46) | 8.79(6.87 to 10.92) | 1.13 (0.92 to 1.35) |
| Belarus | 53.02(38.41 to 72.82) | 93.58(65.34 to 134.8) | 0.43(0.32 to 0.6) | 0.59(0.41 to 0.84) | 1.4 (0.83 to 1.98) |
| Belgium | 300.21(216.25 to 418) | 543.7(367.84 to 805.28) | 1.9(1.38 to 2.6) | 1.86(1.31 to 2.7) | -0.02 (-0.12 to 0.08) |
| Belize | 6.87(5.57 to 8.25) | 37.93(30.77 to 46) | 7.47(6.02 to 9.05) | 13.91(11.2 to 16.88) | 2.36 (1.85 to 2.88) |
| Benin | 199.41(150.55 to 255.43) | 457.47(331.14 to 619.98) | 10.65(8.05 to 13.67) | 10.38(7.56 to 13.87) | 0.07 (-0.03 to 0.17) |
| Bermuda | 3.32(2.65 to 4) | 6.24(4.79 to 7.92) | 5.55(4.43 to 6.66) | 4.67(3.59 to 5.92) | -0.3 (-0.47 to -0.12) |
| Bhutan | 16.17(9.94 to 24.22) | 50.62(34.16 to 68.83) | 7.05(4.31 to 10.6) | 9.64(6.54 to 13.12) | 1.21 (1.14 to 1.28) |
| Bolivia (Plurinational State of) | 377.48(286.67 to 489.08) | 1484.83(1068.8 to 2014.02) | 12.82(9.89 to 16.71) | 18.63(13.62 to 24.99) | 1.37 (1.3 to 1.44) |
| Bosnia and Herzegovina | 74.7(52.09 to 98.33) | 167.78(111.83 to 238.69) | 1.98(1.4 to 2.6) | 2.84(1.93 to 4) | 1.47 (1.16 to 1.79) |
| Botswana | 36.87(23.79 to 54.03) | 134.17(86.32 to 198.25) | 7.27(4.86 to 10.54) | 11.17(7.62 to 16.29) | 1.02 (0.59 to 1.46) |
| Brazil | 4521.11(3750.95 to 5295.72) | 12230.33(9996.03 to 14631.83) | 5.56(4.59 to 6.56) | 5.34(4.36 to 6.41) | -0.12 (-0.21 to -0.03) |
| Brunei Darussalam | 15.79(13.44 to 18.3) | 42.31(35.4 to 49.68) | 19.17(16.3 to 22.39) | 18.88(15.78 to 22.24) | 0.63 (0.37 to 0.88) |
| Bulgaria | 196.97(140.01 to 255.01) | 487.69(335.7 to 683.31) | 1.67(1.22 to 2.14) | 3.43(2.36 to 4.73) | 2.6 (2.24 to 2.97) |
| Burkina Faso | 348.81(256.65 to 455.61) | 779.89(578.51 to 1023.99) | 9.36(7.01 to 12.17) | 9.8(7.36 to 12.81) | 0.11 (0 to 0.22) |
| Burundi | 216.23(154.6 to 289.55) | 294.35(210.74 to 400.86) | 10.03(7.16 to 13.35) | 7.75(5.58 to 10.34) | -1.19 (-1.31 to -1.08) |
| Cabo Verde | 10.89(8.33 to 14.23) | 33.96(26.54 to 42.05) | 4.62(3.56 to 5.9) | 8.07(6.33 to 9.95) | 1.16 (0.84 to 1.47) |
| Cambodia | 541.26(431.54 to 675.86) | 1085.84(844.13 to 1331.79) | 11.09(8.79 to 13.79) | 9.35(7.35 to 11.33) | -0.69 (-0.78 to -0.61) |
| Cameroon | 630.43(456.83 to 826.81) | 1640.67(1175.97 to 2247.84) | 16.08(11.6 to 21.09) | 15.37(11.42 to 20.48) | -0.15 (-0.25 to -0.04) |
| Canada | 637.99(473.17 to 834.47) | 1508.22(1082.72 to 2047.59) | 1.99(1.48 to 2.58) | 1.98(1.45 to 2.7) | -0.43 (-0.6 to -0.25) |
| Central African Republic | 99.57(67.06 to 135.21) | 184.87(124.06 to 260.1) | 9.56(6.62 to 12.66) | 9.73(6.63 to 13.56) | 0.1 (-0.03 to 0.23) |
| Chad | 250.85(175.27 to 371.88) | 507.66(349.48 to 707.43) | 9.51(6.61 to 14.06) | 9.98(6.96 to 13.92) | 0.28 (0.17 to 0.39) |
| Chile | 414.45(312.52 to 513.07) | 1545.04(1190.2 to 1950.73) | 4.54(3.46 to 5.66) | 6.46(5 to 8.16) | 1.52 (1.13 to 1.92) |
| China | 38595.74(31794.16 to 46115.99) | 76033.48(60573.09 to 91453.62) | 4.95(4.13 to 5.82) | 4.22(3.41 to 5.03) | -0.26 (-0.39 to -0.14) |
| Colombia | 1095.98(873.9 to 1308.48) | 3022.04(2180.41 to 4104.79) | 6.8(5.38 to 8.19) | 5.59(4.01 to 7.58) | -0.78 (-0.97 to -0.59) |
| Comoros | 18.66(11.28 to 25.68) | 37.08(27.86 to 48.64) | 9.34(6.01 to 12.71) | 8.43(6.38 to 11.08) | -0.55 (-0.67 to -0.42) |
| Congo | 110.23(71.08 to 152.01) | 220.47(143.74 to 305.77) | 11.89(7.72 to 16.24) | 10.17(6.58 to 14.01) | -0.71 (-0.83 to -0.6) |
| Cook Islands | 1.26(1 to 1.53) | 3.08(2.48 to 3.77) | 10.5(8.55 to 12.7) | 13.14(10.59 to 16.13) | 0.98 (0.89 to 1.06) |
| Costa Rica | 82.83(65.75 to 100.27) | 450.15(325.78 to 614.6) | 4.93(3.88 to 5.99) | 8.73(6.3 to 11.9) | 1.76 (1.08 to 2.44) |
| Croatia | 117.66(85.73 to 154.18) | 231.46(155.57 to 327.86) | 1.93(1.42 to 2.52) | 2.42(1.65 to 3.39) | 0.44 (0.08 to 0.8) |
| Cuba | 255.15(206.72 to 301.91) | 852.96(629.38 to 1139.86) | 2.51(2.03 to 2.97) | 4.47(3.28 to 5.96) | 2.55 (2.31 to 2.79) |
| Cyprus | 54.83(38.45 to 76.26) | 87.56(62.5 to 121.7) | 9.32(6.73 to 12.74) | 5.19(3.73 to 7.21) | -2.35 (-2.5 to -2.19) |
| Czechia | 300.09(222.04 to 385.98) | 336.79(232.18 to 462.76) | 2.19(1.63 to 2.78) | 1.52(1.07 to 2.07) | -1.23 (-1.36 to -1.1) |
| Cte d'Ivoire | 402.67(285.92 to 527.36) | 984.49(689.19 to 1307.08) | 11.71(8.57 to 15.11) | 10.6(7.64 to 13.92) | -0.52 (-0.68 to -0.35) |
| Democratic People's Republic of Korea | 1040.22(773.42 to 1349.05) | 1898.63(1454.26 to 2423.67) | 6.68(5.12 to 8.38) | 6.11(4.7 to 7.79) | -0.28 (-0.4 to -0.15) |
| Democratic Republic of the Congo | 1209.44(903.08 to 1557.24) | 2328.85(1651.24 to 3095.89) | 9.33(7.14 to 12) | 7.68(5.43 to 10.12) | -0.77 (-0.81 to -0.72) |
| Denmark | 92.06(67.71 to 124.49) | 273.02(196.76 to 380.98) | 1.08(0.8 to 1.44) | 2.13(1.55 to 2.93) | 2.12 (1.69 to 2.56) |
| Djibouti | 8.15(5.4 to 11.59) | 43(30.15 to 59.54) | 7.29(5.02 to 10.21) | 9.25(6.6 to 12.29) | 0.83 (0.73 to 0.93) |
| Dominica | 8.73(7.05 to 10.57) | 14.63(11.26 to 18.44) | 12.18(9.89 to 14.67) | 16.32(12.59 to 20.55) | 1.3 (1.19 to 1.41) |
| Dominican Republic | 157.66(125.07 to 193.96) | 693.96(478.79 to 980.65) | 4.45(3.49 to 5.49) | 7.63(5.28 to 10.64) | 2.94 (2.6 to 3.27) |
| Ecuador | 318.36(255.77 to 380.66) | 2238.6(1650.92 to 2964.15) | 6.39(5.14 to 7.63) | 16.16(12.01 to 21.33) | 3.55 (2.88 to 4.23) |
| Egypt | 3203.06(2097.85 to 4234.61) | 8197.45(4662.51 to 12385.69) | 13.02(8.53 to 17.35) | 15.64(9.17 to 23.16) | 0.84 (0.74 to 0.93) |
| El Salvador | 201.89(162.39 to 240.26) | 1443.06(1033.33 to 1918.4) | 6.81(5.45 to 8.12) | 24.05(17.13 to 31.94) | 4.62 (3.87 to 5.38) |
| Equatorial Guinea | 16.04(10.77 to 22.1) | 42.85(29.19 to 62.69) | 9.05(6.18 to 12.37) | 10.82(7.31 to 15.21) | 0.83 (0.7 to 0.96) |
| Eritrea | 68.86(43.93 to 102.22) | 198.8(131.81 to 284.42) | 7.94(4.98 to 11.98) | 9.03(6.03 to 12.74) | 0.36 (0.2 to 0.51) |
| Estonia | 12.87(9.32 to 17.08) | 87.11(59.75 to 124.73) | 0.65(0.48 to 0.86) | 2.86(1.98 to 4.02) | 5.45 (4.85 to 6.06) |
| Eswatini | 29.57(20.81 to 39.62) | 87.86(58.9 to 121.32) | 11.19(8 to 14.94) | 17(11.72 to 22.98) | 1.63 (0.97 to 2.3) |
| Ethiopia | 2317.57(1755.94 to 2910.32) | 3056.22(2444.71 to 3717.69) | 13.21(10.24 to 16.47) | 8.67(6.81 to 10.68) | -1.5 (-1.62 to -1.39) |
| Fiji | 55.96(43.65 to 70.56) | 139.5(106.28 to 176.17) | 15.85(12.52 to 19.94) | 21.11(16.39 to 26.47) | 0.47 (0.09 to 0.85) |
| Finland | 70.33(53.94 to 89.94) | 210.85(148.67 to 283.08) | 0.99(0.77 to 1.26) | 1.41(1.02 to 1.87) | 2.48 (2.09 to 2.87) |
| France | 1397.3(991.96 to 1985.34) | 2423.6(1655.45 to 3525.43) | 1.56(1.14 to 2.16) | 1.3(0.92 to 1.82) | -0.57 (-0.72 to -0.42) |
| Gabon | 58.16(38.96 to 78.82) | 121(71.1 to 167.97) | 11.66(7.74 to 15.76) | 13.72(7.82 to 19.03) | 0.51 (0.28 to 0.74) |
| Gambia | 28.96(20.27 to 39.99) | 90.62(66.95 to 122.14) | 9.39(6.71 to 12.72) | 10.26(7.53 to 13.8) | 0.25 (0.13 to 0.37) |
| Georgia | 114.65(86.15 to 145.21) | 250.19(189.38 to 320.31) | 1.96(1.49 to 2.45) | 4.17(3.15 to 5.34) | 4.25 (3.44 to 5.07) |
| Germany | 2792.71(2066.04 to 3663) | 9964.54(7105.44 to 13622.73) | 2.08(1.56 to 2.68) | 4.18(3.02 to 5.64) | 3.35 (2.98 to 3.72) |
| Ghana | 505.87(356.53 to 705.52) | 1600.62(1088.78 to 2158.74) | 9.14(6.57 to 12.64) | 11.3(7.8 to 15.06) | 0.9 (0.72 to 1.07) |
| Greece | 728.15(536.59 to 992.2) | 1041(731.72 to 1464.11) | 4.94(3.66 to 6.66) | 3.47(2.51 to 4.74) | -1.09 (-1.52 to -0.67) |
| Greenland | 0.9(0.65 to 1.15) | 1.63(1.11 to 2.26) | 3.48(2.57 to 4.58) | 2.83(1.97 to 3.95) | -0.93 (-1.13 to -0.74) |
| Grenada | 8.81(7.19 to 10.56) | 17.62(14.51 to 21.36) | 11.92(9.74 to 14.21) | 16.69(13.83 to 20.18) | 1.56 (1.4 to 1.73) |
| Guam | 6.63(5.46 to 7.82) | 24.61(19.73 to 30.18) | 10.39(8.59 to 12.29) | 13.12(10.56 to 16.1) | 1.1 (0.82 to 1.37) |
| Guatemala | 339.43(268.59 to 410.26) | 2153.77(1603.22 to 2794.87) | 10.48(8.37 to 12.82) | 20.64(15.38 to 26.54) | 3.05 (2.68 to 3.42) |
| Guinea | 325.56(228.41 to 451.81) | 546.36(394.03 to 722.48) | 10.67(7.49 to 14.96) | 10.62(7.69 to 14.03) | -0.1 (-0.2 to -0.01) |
| Guinea-Bissau | 62.27(45.02 to 83.15) | 92.18(66.73 to 123.39) | 16.45(12.12 to 21.6) | 13.75(10.08 to 18.27) | -0.58 (-0.63 to -0.53) |
| Guyana | 36.72(29.1 to 45.37) | 100.47(72.61 to 131.4) | 9.96(7.94 to 12.34) | 16.6(12.14 to 21.36) | 2.48 (2.24 to 2.71) |
| Haiti | 359.09(255.22 to 534.19) | 755.88(495.15 to 1227.7) | 11.4(8.12 to 17.41) | 11.3(7.56 to 17.84) | 0.29 (0.15 to 0.44) |
| Honduras | 176.29(125.75 to 259.17) | 1142.59(835.18 to 1535.89) | 8.76(6.25 to 13.23) | 20.02(14.71 to 26.86) | 3.32 (3.03 to 3.62) |
| Hungary | 236.44(178.12 to 293.8) | 497.96(354.94 to 672.14) | 1.67(1.27 to 2.08) | 2.43(1.74 to 3.28) | 2.44 (2.03 to 2.86) |
| Iceland | 2.08(1.49 to 2.97) | 5.29(3.6 to 7.67) | 0.69(0.49 to 0.97) | 0.81(0.55 to 1.16) | 0.71 (-0.11 to 1.53) |
| India | 26289.23(19325.71 to 33704.67) | 76778.32(58819.13 to 97037.63) | 6.56(4.93 to 8.33) | 7.06(5.45 to 8.89) | 0.14 (-0.14 to 0.41) |
| Indonesia | 9789.34(8142.93 to 11467.5) | 19718.93(15857.31 to 23935.38) | 9.2(7.64 to 10.79) | 9.28(7.55 to 11.02) | 0.05 (-0.07 to 0.17) |
| Iran (Islamic Republic of) | 1636.88(1327.46 to 1948.7) | 4333.52(3525.69 to 5117.23) | 8.04(6.55 to 9.82) | 6.72(5.47 to 7.97) | -0.81 (-0.92 to -0.7) |
| Iraq | 1220.55(926.14 to 1649.2) | 2999.02(2199.14 to 3978.53) | 17.04(12.77 to 23.56) | 15.55(11.47 to 20.63) | -0.41 (-0.47 to -0.35) |
| Ireland | 98.09(71.28 to 135.48) | 155.82(108.82 to 222.55) | 2.5(1.82 to 3.4) | 1.98(1.39 to 2.81) | -0.86 (-0.93 to -0.79) |
| Israel | 282.25(206.35 to 371.01) | 832.22(625.3 to 1097.13) | 6.25(4.64 to 8.23) | 6.55(4.92 to 8.55) | 1.33 (0.52 to 2.15) |
| Italy | 1779.96(1322.42 to 2374.86) | 3175.87(2190.02 to 4455.87) | 2.02(1.52 to 2.67) | 1.68(1.19 to 2.29) | -0.68 (-1.23 to -0.13) |
| Jamaica | 142.31(115.85 to 169.11) | 331.45(250.96 to 428.57) | 7.85(6.43 to 9.29) | 10.74(8.05 to 13.91) | 0.48 (-0.04 to 1.01) |
| Japan | 8080.92(6664.9 to 9459.77) | 16892.76(12450.38 to 21224.02) | 5.18(4.28 to 6.08) | 3.46(2.71 to 4.23) | -1.44 (-1.61 to -1.27) |
| Jordan | 166.56(131.43 to 205.87) | 707.94(545.31 to 884.12) | 15.53(12.2 to 19.48) | 13.65(10.56 to 17.04) | -0.36 (-0.54 to -0.18) |
| Kazakhstan | 280.06(216.46 to 343.87) | 636.55(483.98 to 805.02) | 2.19(1.7 to 2.68) | 3.94(3.01 to 4.96) | 1.6 (1.28 to 1.92) |
| Kenya | 424.42(319.56 to 564.95) | 1345.53(1021.72 to 1699.87) | 6.03(4.5 to 7.98) | 7.52(5.77 to 9.49) | 0.85 (0.77 to 0.93) |
| Kiribati | 8.13(6.37 to 10.12) | 18.37(13.24 to 24.74) | 20.95(16.6 to 25.99) | 26.6(19.86 to 35.41) | 0.53 (0.12 to 0.95) |
| Kuwait | 48.78(39.11 to 58.12) | 114.39(88.29 to 145.61) | 9.7(7.71 to 11.75) | 5.6(4.22 to 7.23) | -2.24 (-3.02 to -1.45) |
| Kyrgyzstan | 110.41(82.93 to 139.94) | 140.6(103.99 to 181.69) | 3.49(2.61 to 4.45) | 2.91(2.17 to 3.69) | -1.22 (-1.66 to -0.78) |
| Lao People's Democratic Republic | 525.96(391.77 to 683.16) | 874.68(653.58 to 1146.95) | 24.79(18.65 to 31.95) | 20.37(15.64 to 26.26) | -0.86 (-0.93 to -0.78) |
| Latvia | 12.98(8.99 to 17.9) | 47.25(32 to 67.46) | 0.37(0.26 to 0.51) | 1.11(0.76 to 1.55) | 4.98 (4.42 to 5.54) |
| Lebanon | 218.72(167.18 to 281.04) | 389.54(269.19 to 537.93) | 11.08(8.55 to 14.29) | 7.61(5.27 to 10.45) | -1.25 (-1.42 to -1.09) |
| Lesotho | 58.97(40.26 to 81.8) | 173.95(114.58 to 239.97) | 6.52(4.51 to 8.9) | 15.33(10.3 to 20.7) | 3.62 (3.26 to 3.98) |
| Liberia | 129.05(94.02 to 170.05) | 197.5(134.27 to 278.54) | 12.87(9.46 to 16.79) | 10.72(7.45 to 15.18) | -0.4 (-0.68 to -0.11) |
| Libya | 173.02(121.09 to 234.44) | 486.6(311.58 to 688.13) | 10.19(7.1 to 14.04) | 10.55(6.69 to 15.06) | 0.32 (0.16 to 0.49) |
| Lithuania | 20.76(14.75 to 28.38) | 48.24(32.65 to 69.04) | 0.47(0.33 to 0.63) | 0.8(0.54 to 1.11) | 1.38 (0.77 to 2) |
| Luxembourg | 12.57(9.05 to 17.45) | 27.11(18.76 to 39.13) | 2.38(1.74 to 3.29) | 2.36(1.66 to 3.36) | 0.2 (-0.01 to 0.42) |
| Madagascar | 312.07(224.27 to 425.96) | 562(388.2 to 786.07) | 6.8(4.88 to 9.23) | 6.29(4.38 to 8.8) | -0.37 (-0.46 to -0.28) |
| Malawi | 273.02(204.48 to 345.56) | 517.8(387.69 to 663.34) | 8.37(6.32 to 10.64) | 8.34(6.23 to 10.62) | -0.16 (-0.32 to 0) |
| Malaysia | 1097.65(939.32 to 1251.95) | 3047.04(2349.55 to 3834.12) | 12.07(10.24 to 13.84) | 12.24(9.48 to 15.31) | -0.52 (-0.77 to -0.27) |
| Maldives | 21.8(17.88 to 26.81) | 41.73(33.17 to 51.01) | 26.99(22.37 to 33.2) | 15.11(11.89 to 18.84) | -2.42 (-2.69 to -2.15) |
| Mali | 412.65(299.01 to 547.74) | 760.84(552.56 to 1019.3) | 11.2(8.25 to 14.98) | 9.87(7.32 to 12.97) | -0.26 (-0.42 to -0.09) |
| Malta | 13.17(9.52 to 17.89) | 24.78(17.55 to 34.52) | 3.35(2.47 to 4.54) | 2.43(1.74 to 3.35) | -1.08 (-1.31 to -0.86) |
| Marshall Islands | 2.96(2.28 to 3.9) | 8.07(5.67 to 11.32) | 17.73(13.63 to 23.67) | 23.92(17.19 to 32.92) | 1.02 (0.76 to 1.28) |
| Mauritania | 140.32(103.83 to 181.17) | 188.84(131.32 to 254.02) | 15.22(11.37 to 19.7) | 10.09(7.15 to 13.5) | -1.38 (-1.47 to -1.29) |
| Mauritius | 161.24(140.36 to 180.7) | 660.59(525.23 to 821.25) | 22.3(19.35 to 25.07) | 38.72(30.87 to 47.8) | 2.24 (1.87 to 2.61) |
| Mexico | 3708.52(3131.43 to 4286.19) | 23439.97(18624.19 to 28802.78) | 9.66(8.08 to 11.26) | 20.5(16.31 to 24.91) | 2.91 (2.47 to 3.35) |
| Micronesia (Federated States of) | 10.44(7.96 to 13.51) | 23.63(16.03 to 32.09) | 22.47(17.44 to 29.07) | 35.21(25.54 to 46.8) | 1.45 (1.05 to 1.85) |
| Monaco | 0.79(0.53 to 1.16) | 1.71(1.17 to 2.39) | 0.96(0.66 to 1.37) | 1.43(0.99 to 1.99) | 1.79 (1.34 to 2.23) |
| Mongolia | 101.28(74.14 to 131.25) | 116.87(81.58 to 161.87) | 9.97(7.35 to 13.03) | 5.32(3.86 to 7.19) | -3.38 (-3.85 to -2.9) |
| Montenegro | 19.5(14.62 to 25.42) | 35.8(25.37 to 48.07) | 3.27(2.42 to 4.26) | 3.73(2.66 to 4.99) | 0.4 (0.26 to 0.54) |
| Morocco | 1123.68(817.27 to 1539.27) | 3174.17(2260.42 to 4291.69) | 9.32(6.75 to 13.11) | 11.7(8.41 to 16.01) | 1.09 (0.94 to 1.24) |
| Mozambique | 341.52(241.23 to 462.01) | 742.85(534.23 to 1002.04) | 6.91(4.93 to 9.36) | 8.13(5.89 to 10.9) | 0.59 (0.47 to 0.72) |
| Myanmar | 3574.81(2671.28 to 4636.61) | 5675(4514.14 to 7217.26) | 14.52(10.99 to 18.65) | 12.66(10.31 to 15.82) | -0.54 (-0.66 to -0.42) |
| Namibia | 46.69(30.47 to 70.27) | 92.17(60.65 to 138) | 7.19(4.8 to 10.59) | 7.24(4.84 to 10.91) | -0.41 (-0.85 to 0.03) |
| Nauru | 0.89(0.63 to 1.16) | 1.31(0.87 to 1.76) | 22.28(16.69 to 28.4) | 30.03(21.33 to 38.47) | 0.91 (0.59 to 1.23) |
| Nepal | 482.58(309.41 to 689.53) | 1732.96(1158.72 to 2427) | 5.38(3.51 to 7.69) | 8.34(5.49 to 11.66) | 1.71 (1.39 to 2.03) |
| Netherlands | 313.46(225.48 to 445.85) | 690.53(477.5 to 976.83) | 1.55(1.12 to 2.17) | 1.8(1.25 to 2.52) | 0.75 (0.41 to 1.1) |
| New Zealand | 40.42(28.38 to 56.08) | 128.44(92.11 to 176.1) | 1.08(0.77 to 1.5) | 1.54(1.11 to 2.1) | 1.47 (1.07 to 1.87) |
| Nicaragua | 190.4(152.12 to 229.16) | 1227.98(935.18 to 1563.47) | 12.67(10.04 to 15.45) | 29.22(22.54 to 36.83) | 3.28 (2.93 to 3.62) |
| Niger | 255.73(181.11 to 350.62) | 594.54(417.03 to 810.69) | 10.45(7.48 to 14.29) | 8.87(6.41 to 11.97) | -0.46 (-0.54 to -0.37) |
| Nigeria | 2910.28(2063.16 to 4006.9) | 5154.63(3664.16 to 6911.25) | 7.71(5.5 to 10.48) | 7.06(5.12 to 9.22) | -0.58 (-0.76 to -0.41) |
| Niue | 0.36(0.27 to 0.45) | 0.44(0.31 to 0.57) | 16.07(12.27 to 20.43) | 21.11(14.86 to 27.04) | 0.81 (0.55 to 1.07) |
| North Macedonia | 40.4(28.37 to 53.61) | 85.07(56.13 to 120.96) | 2.26(1.6 to 3.02) | 2.79(1.9 to 3.89) | 0.37 (-0.01 to 0.75) |
| Northern Mariana Islands | 3.59(2.79 to 4.6) | 11.06(8.67 to 13.59) | 19.89(16.09 to 24.85) | 24.43(19.74 to 29.63) | 0.91 (0.8 to 1.02) |
| Norway | 66.12(47.56 to 91.78) | 156.87(111.65 to 213.27) | 0.88(0.64 to 1.2) | 1.36(0.98 to 1.83) | 1.74 (1.55 to 1.93) |
| Oman | 30(21.26 to 41.71) | 75.86(58.9 to 93.57) | 5.96(4.3 to 8.22) | 7.15(5.49 to 8.98) | 1.01 (0.84 to 1.18) |
| Pakistan | 4197.09(2868.45 to 6081.26) | 13148.59(9157.06 to 17947.99) | 7.69(5.27 to 11.24) | 12.43(8.87 to 16.76) | 1.67 (1.4 to 1.95) |
| Palau | 2.37(1.85 to 3.1) | 6.09(4.61 to 7.9) | 24.63(19.53 to 31.98) | 31.44(24.27 to 39.95) | 0.79 (0.59 to 0.99) |
| Palestine | 126.22(92.68 to 168.45) | 234.39(184.04 to 294.49) | 15.93(11.65 to 21.15) | 11.91(9.41 to 14.78) | -0.7 (-0.98 to -0.43) |
| Panama | 70.65(56.86 to 84.3) | 393.12(288.01 to 520.71) | 4.83(3.86 to 5.78) | 9.38(6.83 to 12.48) | 2.35 (1.98 to 2.72) |
| Papua New Guinea | 109.29(83.54 to 138.05) | 332.32(247.2 to 437.4) | 5.38(4.15 to 6.75) | 6.34(4.75 to 8.14) | 0.49 (0.35 to 0.64) |
| Paraguay | 107.95(86.86 to 129.85) | 659.97(483.9 to 861.36) | 5.09(4.05 to 6.17) | 12.21(9.02 to 15.89) | 3.73 (3.48 to 3.98) |
| Peru | 792.75(611.01 to 988.65) | 2423.7(1688.3 to 3331.08) | 7(5.41 to 8.76) | 7.51(5.22 to 10.35) | 0.34 (0.12 to 0.55) |
| Philippines | 4589.58(3894.88 to 5327.16) | 15631.88(12443.77 to 19156.58) | 16.35(13.88 to 19.2) | 20.16(16.16 to 24.6) | 1.12 (0.86 to 1.39) |
| Poland | 1170.17(898.4 to 1430.28) | 1236.12(920.63 to 1611.12) | 2.74(2.13 to 3.32) | 1.7(1.28 to 2.2) | -1.4 (-1.97 to -0.81) |
| Portugal | 381.82(277.74 to 510.78) | 934.41(651.98 to 1313.36) | 2.92(2.16 to 3.86) | 3.14(2.27 to 4.34) | 0.38 (-0.1 to 0.87) |
| Puerto Rico | 345.3(282.13 to 406.1) | 715.96(517.82 to 933.7) | 9.82(8.09 to 11.5) | 9.41(6.83 to 12.26) | 0.44 (0.13 to 0.75) |
| Qatar | 11.1(8.28 to 17.06) | 53.63(38.86 to 72.84) | 17.93(13.18 to 30.59) | 16.53(12.53 to 21.74) | 0.02 (-0.26 to 0.31) |
| Republic of Korea | 1546.66(1403.9 to 1703.44) | 4232.11(3579.76 to 4867.37) | 5.9(5.21 to 6.64) | 4.96(4.19 to 5.71) | -0.4 (-0.56 to -0.24) |
| Republic of Moldova | 21.37(15.64 to 28.21) | 52.52(36.65 to 69.76) | 0.58(0.43 to 0.77) | 0.92(0.65 to 1.22) | 1.77 (1.41 to 2.14) |
| Romania | 307.67(182.7 to 418.16) | 525.82(349.85 to 755.37) | 1.2(0.73 to 1.61) | 1.36(0.92 to 1.92) | 1.19 (0.64 to 1.74) |
| Russian Federation | 1833.77(1339.25 to 2369.83) | 2466.54(1796.66 to 3232.33) | 1.07(0.8 to 1.39) | 1.07(0.78 to 1.37) | -0.39 (-0.69 to -0.09) |
| Rwanda | 270.48(201.75 to 346.44) | 387.15(281.07 to 507.39) | 10.49(7.89 to 13.48) | 7.89(5.68 to 10.32) | -1.74 (-2.06 to -1.42) |
| Saint Kitts and Nevis | 5.94(4.74 to 7.19) | 11.13(8.36 to 14) | 16.6(13.58 to 19.71) | 18.33(14.15 to 22.78) | 0.71 (0.53 to 0.88) |
| Saint Lucia | 9.11(7.51 to 10.76) | 24.32(19 to 29.75) | 10.95(9.06 to 12.93) | 11.63(9.12 to 14.22) | 0.26 (0.03 to 0.5) |
| Saint Vincent and the Grenadines | 6.2(5.11 to 7.33) | 16.55(13.29 to 20.32) | 8.88(7.4 to 10.5) | 12.86(10.4 to 15.68) | 1.57 (1.34 to 1.8) |
| Samoa | 14.15(10.53 to 18.69) | 28.06(21.65 to 36.12) | 16.89(12.76 to 22.25) | 19.84(15.47 to 25.24) | 0.47 (0.26 to 0.68) |
| San Marino | 0.3(0.21 to 0.42) | 0.94(0.57 to 1.51) | 0.92(0.65 to 1.31) | 1.15(0.7 to 1.81) | 1.5 (1.24 to 1.77) |
| Sao Tome and Principe | 7.27(5.36 to 9.42) | 14.68(10.64 to 19.38) | 12.52(9.32 to 16.18) | 15.5(11.34 to 20.34) | 0.65 (0.52 to 0.78) |
| Saudi Arabia | 864.55(623.92 to 1178.51) | 2545.5(1857.68 to 3376.57) | 16.73(12.12 to 22.65) | 18.13(13.39 to 23.75) | 0.51 (0.26 to 0.77) |
| Senegal | 376.82(268.61 to 509.54) | 776.37(552.28 to 1057.96) | 13.02(9.5 to 17.47) | 11.53(8.37 to 15.61) | -0.34 (-0.47 to -0.21) |
| Serbia | 375.21(267.93 to 498.27) | 654.4(446.05 to 913.93) | 3.59(2.56 to 4.8) | 4.05(2.81 to 5.63) | -0.03 (-0.21 to 0.16) |
| Seychelles | 7.97(6.67 to 9.22) | 21.46(17.86 to 25.1) | 14.14(11.85 to 16.38) | 20.91(17.38 to 24.49) | 0.87 (0.49 to 1.25) |
| Sierra Leone | 167.83(121.64 to 222.02) | 290.84(209.83 to 393.28) | 9.41(6.87 to 12.41) | 8.82(6.45 to 11.79) | -0.02 (-0.11 to 0.08) |
| Singapore | 158.58(138.07 to 179.08) | 368.28(303.02 to 431.72) | 7.84(6.81 to 8.86) | 5.02(4.09 to 5.89) | -0.02 (-0.49 to 0.46) |
| Slovakia | 211.41(157.52 to 264.83) | 218.98(148.38 to 303.1) | 3.57(2.66 to 4.44) | 2.38(1.62 to 3.27) | -0.14 (-0.65 to 0.38) |
| Slovenia | 30.52(21.7 to 41.76) | 50.34(32.99 to 73.98) | 1.29(0.93 to 1.75) | 0.99(0.66 to 1.45) | -0.42 (-0.64 to -0.2) |
| Solomon Islands | 32.24(22.95 to 44.04) | 44.87(34.57 to 56.74) | 19.73(14.16 to 26.89) | 11.94(9.45 to 14.7) | -1.91 (-2.32 to -1.49) |
| Somalia | 219.44(150.01 to 305.83) | 536.09(370.63 to 758.01) | 10.08(7.16 to 13.89) | 9.64(6.73 to 13.17) | 0 (-0.04 to 0.05) |
| South Africa | 1103.58(846.26 to 1397.86) | 3485.66(2685.44 to 4310.38) | 5.51(4.25 to 7.05) | 8.79(6.85 to 10.82) | 2.33 (1.98 to 2.68) |
| South Sudan | 181.83(124.08 to 262.64) | 272.89(184.37 to 385.87) | 8.62(5.9 to 12.33) | 8.64(5.91 to 11.87) | 0.01 (-0.01 to 0.04) |
| Spain | 1567.29(1114.47 to 2134.78) | 2530.24(1669.22 to 3768.45) | 2.94(2.11 to 4.01) | 1.97(1.35 to 2.86) | -1.49 (-1.63 to -1.34) |
| Sri Lanka | 1205.92(1010.68 to 1402.22) | 2759.88(2008.6 to 3726.92) | 11.67(9.76 to 13.59) | 11.63(8.51 to 15.5) | 0.09 (-0.3 to 0.48) |
| Sudan | 714.04(487.16 to 1054.71) | 1449.24(910.08 to 2271.33) | 8.39(5.61 to 12.32) | 8.84(5.54 to 13.93) | -0.12 (-0.36 to 0.12) |
| Suriname | 24.1(19.82 to 28.74) | 90.94(70.62 to 111.97) | 9.43(7.69 to 11.28) | 15.63(12.18 to 19.27) | 1.82 (1.53 to 2.12) |
| Sweden | 184.99(143.54 to 234.34) | 560.15(415.83 to 717.93) | 1.11(0.87 to 1.39) | 2.12(1.61 to 2.69) | 2.31 (2.14 to 2.49) |
| Switzerland | 170.77(124.93 to 228.46) | 552.39(376.67 to 770.41) | 1.51(1.12 to 1.99) | 2.43(1.69 to 3.32) | 2.01 (1.84 to 2.17) |
| Syrian Arab Republic | 555.82(412.95 to 728.75) | 886.56(626.19 to 1221.49) | 11.6(8.59 to 15.44) | 8.72(6.32 to 11.74) | -1.64 (-1.94 to -1.33) |
| Taiwan (Province of China) | 1523.68(1322.77 to 1726.82) | 3449.27(2612.6 to 4446.99) | 11.43(9.91 to 13.02) | 8.59(6.45 to 11.1) | -1.11 (-1.28 to -0.94) |
| Tajikistan | 27.17(21.02 to 33.77) | 121.88(89.95 to 161.98) | 0.93(0.72 to 1.18) | 2.98(2.22 to 3.95) | 4.52 (4.14 to 4.91) |
| Thailand | 4819.89(4061.12 to 5690.6) | 12636.15(9211.5 to 16493.6) | 13.77(11.62 to 16.22) | 12.75(9.31 to 16.69) | -0.23 (-0.36 to -0.11) |
| Timor-Leste | 43.67(31.96 to 60.69) | 112.48(81.51 to 148.54) | 14.77(10.99 to 20.41) | 14.67(10.84 to 19.1) | -0.04 (-0.26 to 0.17) |
| Togo | 115.17(83.82 to 155.2) | 305.88(218.16 to 412.52) | 10.35(7.65 to 13.98) | 9.56(6.99 to 12.74) | -0.27 (-0.32 to -0.22) |
| Tokelau | 0.19(0.14 to 0.26) | 0.23(0.17 to 0.32) | 14.92(11.18 to 20.08) | 18.1(13.54 to 24.7) | 0.64 (0.41 to 0.87) |
| Tonga | 6.79(5.27 to 9.19) | 14.6(11.13 to 19.9) | 12.91(10.17 to 17.37) | 18.57(14.12 to 25.23) | 1.22 (0.85 to 1.59) |
| Trinidad and Tobago | 56(45.1 to 66.9) | 203.46(141.03 to 279.59) | 7.02(5.68 to 8.39) | 11.14(7.74 to 15.21) | 2 (1.76 to 2.25) |
| Tunisia | 299.4(220.75 to 394.61) | 799.86(535.63 to 1133.37) | 6.96(5.2 to 9.2) | 6.95(4.7 to 9.91) | 0.06 (-0.05 to 0.16) |
| Turkey | 4034.45(3033.38 to 5748.83) | 6780.91(5164.85 to 8703.18) | 12.52(9.34 to 18.38) | 8.16(6.2 to 10.54) | -0.92 (-1.2 to -0.65) |
| Turkmenistan | 82.92(63.78 to 101.32) | 236.39(171.33 to 316.67) | 4.15(3.22 to 5.08) | 5.9(4.33 to 7.78) | 0.63 (0.29 to 0.98) |
| Tuvalu | 1.15(0.87 to 1.52) | 2.1(1.5 to 2.93) | 17.51(13.39 to 22.87) | 21.86(15.79 to 30.13) | 0.76 (0.54 to 0.97) |
| Uganda | 460.82(329.72 to 626.69) | 1007.32(729.09 to 1354.65) | 8.27(5.93 to 11.24) | 8.52(6.25 to 11.5) | -0.17 (-0.3 to -0.03) |
| Ukraine | 283.61(208.27 to 380.01) | 500.52(362.18 to 681.62) | 0.44(0.33 to 0.59) | 0.7(0.5 to 0.94) | 1.96 (1.66 to 2.26) |
| United Arab Emirates | 76.06(53.73 to 100.79) | 583.75(358.08 to 963.54) | 22.36(13.88 to 29.48) | 17.58(10.45 to 28.29) | -0.8 (-1.38 to -0.21) |
| United Kingdom | 1034.18(762.29 to 1399.05) | 1481.17(1058.89 to 2062.93) | 1.1(0.81 to 1.48) | 1.01(0.74 to 1.39) | 0.2 (-0.19 to 0.59) |
| United Republic of Tanzania | 644.19(463.06 to 859.69) | 1502.65(1179.48 to 1883.25) | 6.9(5.02 to 9.25) | 7.3(5.6 to 9.25) | 0.2 (0.13 to 0.27) |
| United States of America | 6848.99(5057.87 to 9010.07) | 34937.68(26933.89 to 42941.77) | 2.08(1.54 to 2.7) | 5.91(4.6 to 7.22) | 3.8 (3.38 to 4.22) |
| United States Virgin Islands | 5.75(4.54 to 7.18) | 18.19(13.98 to 22.18) | 7.22(5.76 to 8.98) | 10.34(8.06 to 12.48) | 1.83 (1.58 to 2.07) |
| Uruguay | 144.93(130.81 to 159.73) | 310.21(252.49 to 379.21) | 3.7(3.34 to 4.08) | 5.15(4.26 to 6.22) | 1.66 (1.39 to 1.92) |
| Uzbekistan | 539.39(378.55 to 801.77) | 1309.35(977.07 to 1665.73) | 4.66(3.28 to 7.07) | 6.82(5.17 to 8.47) | 1.01 (0.18 to 1.85) |
| Vanuatu | 7.25(4.89 to 10.45) | 32.02(22.65 to 44.63) | 11.22(7.74 to 15.89) | 19.06(13.9 to 26.04) | 1.97 (1.84 to 2.11) |
| Venezuela (Bolivarian Republic of) | 531.98(435.03 to 623.42) | 3780.29(2690.86 to 5117.82) | 5.71(4.64 to 6.73) | 13.25(9.42 to 17.94) | 2.48 (1.99 to 2.98) |
| Viet Nam | 5071.54(3895.95 to 6420.63) | 10120.89(7574.5 to 12881.98) | 13.19(10.18 to 16.63) | 12.02(9.14 to 15.19) | -0.4 (-0.89 to 0.09) |
| Yemen | 348.6(229.94 to 520.89) | 876.78(609.56 to 1227.82) | 8.02(5.44 to 11.98) | 7.59(5.35 to 10.42) | -0.24 (-0.32 to -0.15) |
| Zambia | 252.97(185.78 to 325) | 584.09(427.98 to 774.05) | 10.21(7.52 to 13.32) | 10.19(7.49 to 13.51) | -0.28 (-0.49 to -0.06) |
| Zimbabwe | 209.63(139.45 to 341.67) | 612.82(394.43 to 958.01) | 6.01(4.04 to 9.85) | 10.03(6.64 to 15.85) | 2.03 (1.63 to 2.43) |

ASMR, age-standard morality rate; EAPC, estimated annual percentage change.
