# Supplementary material for: Global, regional, and national burden of chronic kidney disease attributable to high fasting plasma glucose from 1990 to 2019: a systematic analysis from the global burden of disease study 2019
Source: Front Endocrinol (Lausanne). 2024 Mar 27;15:1379634. doi: 10.3389/fendo.2024.1379634 (PMC11004380; doi:10.3389/fendo.2024.1379634)
Supplement: Supplementary file 4 [file Table_2.docx]

Table S2. DALYs number and age-standardized DALYs rate of chronic kidney disease attributable to high fasting plasma glucose for both sexes combined in 1990 and 2019, and EAPC of ASMR from 1990 to 2019 in 204 countries and territories

| Location | DALYs number in 1990 | DALYs number in 2019 | ASDR in 1990 | ASDR in 2019 | EAPC 1990-2019 |
| --- | --- | --- | --- | --- | --- |
| Afghanistan | 34822.54(24791.96 to 49121.54) | 59875.13(43171.49 to 82960.04) | 475.51(339.84 to 668.03) | 422.55(301.12 to 594.41) | -0.31 (-0.36 to -0.26) |
| Albania | 1130.65(845.61 to 1460.99) | 1741.07(1269.38 to 2364.25) | 50.25(37.87 to 64.71) | 43.09(31.66 to 58.2) | -0.98 (-1.25 to -0.71) |
| Algeria | 31155.22(22318.4 to 45813.91) | 72258.13(53817.2 to 96522.88) | 257.47(187.6 to 373.35) | 217.45(162.1 to 291.71) | -0.44 (-0.61 to -0.26) |
| American Samoa | 123.18(100.46 to 149.12) | 338.51(272.59 to 411.04) | 451.64(369.71 to 541.09) | 677.65(548.16 to 825.11) | 1.47 (1.35 to 1.6) |
| Andorra | 23.91(17.32 to 33.06) | 59.3(43.43 to 79.18) | 46.63(34.23 to 64.29) | 41.54(30.56 to 55.24) | -0.38 (-0.5 to -0.25) |
| Angola | 7932.42(5389.04 to 10934.2) | 20625.75(13614.16 to 28027.29) | 179.67(121.81 to 238.17) | 166.3(111.53 to 221.73) | -0.34 (-0.38 to -0.29) |
| Antigua and Barbuda | 121.8(101.56 to 143.97) | 324.88(260.28 to 396.15) | 234.91(194.68 to 279.22) | 315.62(253.42 to 383.05) | 1.28 (1.12 to 1.44) |
| Argentina | 57586.13(45955.2 to 68309.95) | 105400.74(84807.86 to 126998.8) | 178.95(143.54 to 211.54) | 197.3(158.55 to 237.54) | 0.26 (-0.01 to 0.52) |
| Armenia | 790.34(638.64 to 956.36) | 3970.23(3152.47 to 4887.75) | 29.11(23.58 to 34.82) | 98.01(77.72 to 120.67) | 4.24 (3.9 to 4.58) |
| Australia | 3802.61(3042.43 to 4744.76) | 11863.42(8999.64 to 15303.14) | 19.89(15.89 to 24.79) | 29.07(22.12 to 37.27) | 1.63 (1.44 to 1.83) |
| Austria | 4165.42(3214.71 to 5255.09) | 11871.21(9170.01 to 15314.07) | 35.66(27.77 to 44.28) | 62.65(48.48 to 79.54) | 2.72 (2.24 to 3.2) |
| Azerbaijan | 5106.17(3924.97 to 6329.13) | 15180.57(11795.91 to 18886.21) | 91.48(71.59 to 111.44) | 153.25(121.51 to 188.98) | 2.17 (1.79 to 2.56) |
| Bahamas | 401.7(331.93 to 479.15) | 1150.15(884.96 to 1465.15) | 232.13(191.2 to 275.56) | 279.84(219.37 to 352.99) | 0.92 (0.81 to 1.02) |
| Bahrain | 468.74(370.46 to 587.31) | 2166.84(1681.73 to 2737.16) | 274.57(218.91 to 344.74) | 242.28(191.03 to 304.87) | -0.42 (-0.88 to 0.04) |
| Bangladesh | 50994.99(37024.87 to 66716.73) | 118826.64(89562.25 to 153107.96) | 101.7(73.66 to 131.72) | 89.29(67.65 to 114.78) | -0.02 (-0.27 to 0.23) |
| Barbados | 456.3(383.39 to 527.55) | 1049.89(831.93 to 1288.72) | 167.88(140.59 to 194.41) | 226.47(180.46 to 276.4) | 1.02 (0.85 to 1.19) |
| Belarus | 1981.92(1524.53 to 2497.25) | 3474.5(2643.62 to 4559.72) | 15.78(12.21 to 19.85) | 23.62(17.91 to 30.97) | 1.84 (1.38 to 2.29) |
| Belgium | 6784.83(5189.52 to 8626.19) | 9515.95(7258.09 to 12456.26) | 44.37(34.04 to 55.75) | 40.21(30.99 to 51.92) | -0.31 (-0.39 to -0.22) |
| Belize | 191.54(158.23 to 225.92) | 1159.13(938.23 to 1398.21) | 194.93(159.8 to 230.63) | 374.93(304.85 to 448.98) | 2.39 (1.94 to 2.84) |
| Benin | 5192.31(3927.33 to 6586.54) | 13051.57(9295.26 to 17778.17) | 241.07(182.94 to 303.88) | 235.38(172.16 to 316.94) | 0.07 (-0.02 to 0.16) |
| Bermuda | 90.56(73.63 to 107.24) | 153.78(124.6 to 189.15) | 142.54(115.54 to 168.56) | 127.8(103.23 to 156.72) | -0.15 (-0.29 to -0.01) |
| Bhutan | 506.96(320.02 to 735.85) | 1349.33(936.21 to 1824.31) | 182.23(117.36 to 264.86) | 231.04(161.07 to 309.45) | 0.92 (0.86 to 0.98) |
| Bolivia (Plurinational State of) | 10202.47(7859.31 to 13192.15) | 34693.53(25231.95 to 46158.24) | 298.26(230.1 to 384.32) | 389.88(284.2 to 519.77) | 0.97 (0.9 to 1.04) |
| Bosnia and Herzegovina | 2447.11(1796.79 to 3176.6) | 4337.38(3135.58 to 5733.57) | 57.07(42.51 to 73.28) | 74.69(55.13 to 98.78) | 1.11 (0.86 to 1.37) |
| Botswana | 1090.85(720.48 to 1585.96) | 4062.42(2631.72 to 5945.41) | 177.14(118.81 to 254.5) | 265.59(176.33 to 380.53) | 0.9 (0.44 to 1.37) |
| Brazil | 141249.33(116990.96 to 165061.5) | 317563.39(261992.59 to 374115.51) | 144.5(120.99 to 168.28) | 132.95(110.53 to 156.37) | -0.44 (-0.56 to -0.32) |
| Brunei Darussalam | 471.54(403.35 to 547.64) | 1188.06(1007.06 to 1378) | 417.58(357.43 to 483.27) | 379.89(321.04 to 438.38) | 0.22 (-0.06 to 0.5) |
| Bulgaria | 6497.77(4896.81 to 8101.72) | 12973.1(9407.81 to 17227.97) | 53.7(41.4 to 67.44) | 101.45(73.77 to 136.24) | 2.24 (1.96 to 2.53) |
| Burkina Faso | 9223.83(6891.25 to 11967.8) | 22026.54(16319.24 to 29117.11) | 203.9(154.93 to 263.96) | 218.54(164.74 to 283.34) | 0.17 (0.04 to 0.3) |
| Burundi | 5862.19(4308.25 to 7920.13) | 8525.81(6095.44 to 11490.47) | 233(171.84 to 309.6) | 173.55(127.37 to 228.32) | -1.36 (-1.49 to -1.23) |
| Cabo Verde | 258.85(204.34 to 320.79) | 773.91(611.97 to 947.76) | 113.91(90.32 to 140.75) | 173.88(137.89 to 210.87) | 0.9 (0.68 to 1.13) |
| Cambodia | 20278.81(15990.42 to 25349.61) | 36346.55(28393.69 to 44946.28) | 340.74(272.18 to 423.15) | 266.37(211.21 to 326.02) | -0.98 (-1.06 to -0.9) |
| Cameroon | 17919.58(12956.93 to 23310.72) | 49534.72(34573.6 to 68945.85) | 364.91(268.77 to 475.85) | 354.08(254.75 to 483.05) | -0.12 (-0.28 to 0.04) |
| Canada | 15766.21(12381.31 to 19399.38) | 32208.87(25031.49 to 40525.45) | 48.67(38.54 to 59.82) | 48.05(37.51 to 60.01) | -0.15 (-0.24 to -0.05) |
| Central African Republic | 3041.35(2074.42 to 4149.42) | 5858.54(3904.36 to 8341.38) | 231.93(160.63 to 312.34) | 233.99(160.94 to 323.38) | 0.06 (-0.07 to 0.19) |
| Chad | 6519.95(4583.94 to 9478.95) | 14597.72(10028.19 to 20539.98) | 218.33(153.15 to 317.54) | 230.27(158.96 to 321.24) | 0.31 (0.2 to 0.43) |
| Chile | 10705.69(8374.78 to 12864.49) | 31644.33(25077.06 to 38513.38) | 106.1(83.09 to 127.6) | 132.43(105.32 to 161.32) | 0.96 (0.62 to 1.31) |
| China | 1357346.94(1122111.38 to 1601069.94) | 2134090.93(1763498.54 to 2538658.66) | 142.3(118.61 to 167.14) | 108.93(90.44 to 128.4) | -0.62 (-0.75 to -0.49) |
| Colombia | 32514.79(26597.53 to 37988.99) | 75395.04(57779.11 to 97762.03) | 169.49(137.64 to 198.25) | 142.73(109.25 to 185.24) | -0.8 (-0.97 to -0.62) |
| Comoros | 468.24(259.05 to 648.51) | 912.81(683.5 to 1202.62) | 204.29(119.43 to 280.66) | 183.44(138.35 to 237.93) | -0.6 (-0.77 to -0.43) |
| Congo | 3199.66(2087.27 to 4390.38) | 6351.4(4188.6 to 8777.12) | 276.06(179.6 to 379.56) | 221.81(147.04 to 303.48) | -0.96 (-1.09 to -0.83) |
| Cook Islands | 39.06(31.37 to 48.24) | 81.47(66.37 to 98.5) | 284.63(229.18 to 347.6) | 350.9(283.66 to 427.5) | 0.93 (0.84 to 1.02) |
| Costa Rica | 2705.99(2236.97 to 3183.34) | 11950.75(9095.14 to 15358.2) | 148.72(122.06 to 176.05) | 230.19(176.74 to 296.39) | 1.31 (0.77 to 1.86) |
| Croatia | 3404.65(2588.08 to 4306.27) | 5166.14(3838.39 to 6721.21) | 53.42(40.94 to 67.18) | 60.06(45.41 to 77.78) | 0.11 (-0.18 to 0.4) |
| Cuba | 8346.63(6916.97 to 9783.83) | 23149.11(17785.23 to 29494.32) | 80.18(66.47 to 93.94) | 128.9(98.9 to 163.74) | 1.95 (1.77 to 2.14) |
| Cyprus | 1028.7(770.2 to 1352.68) | 1545.34(1188.89 to 2015.56) | 142.32(108.69 to 188.31) | 82.97(64.06 to 106.46) | -2.18 (-2.28 to -2.07) |
| Czechia | 8402.87(6386.59 to 10367.68) | 8823.51(6661.72 to 11341.09) | 62.38(47.9 to 77.11) | 43.91(33.61 to 56.04) | -1.17 (-1.25 to -1.08) |
| C么te d'Ivoire | 12605.9(8849.39 to 16719.89) | 30207.18(20876.64 to 41120.74) | 267.51(193.89 to 347.5) | 243.14(173.49 to 321.5) | -0.5 (-0.69 to -0.3) |
| Democratic People's Republic of Korea | 38048.37(28884.35 to 49509.6) | 62037.95(48218.28 to 79197.78) | 209.98(162.8 to 266.89) | 191.64(149.44 to 243.99) | -0.3 (-0.38 to -0.23) |
| Democratic Republic of the Congo | 35281.77(26499.5 to 45870.03) | 67627.18(49064.16 to 88434.16) | 207.33(157.63 to 263.61) | 172.24(124.19 to 226.03) | -0.74 (-0.79 to -0.69) |
| Denmark | 2641.66(2050.65 to 3275.77) | 5419.49(4228.94 to 6952.21) | 33.89(26.37 to 42.2) | 47.9(37.59 to 60.5) | 0.93 (0.57 to 1.28) |
| Djibouti | 255.38(172.94 to 361.81) | 1257.25(875.03 to 1808.44) | 161.97(111.47 to 224.62) | 199.02(144.37 to 269.98) | 0.7 (0.6 to 0.81) |
| Dominica | 207.51(170.5 to 248.56) | 350.88(275.1 to 435.63) | 308.75(253.59 to 370.41) | 404.99(315.41 to 503.75) | 1.13 (1.04 to 1.22) |
| Dominican Republic | 5090.19(4050.14 to 6221.01) | 20190.82(14342.5 to 28267) | 121.44(97.93 to 148.03) | 207.84(148.03 to 289.59) | 2.67 (2.39 to 2.94) |
| Ecuador | 8829.79(7201.5 to 10439.88) | 51280.87(38209.3 to 66971.5) | 153.38(124.45 to 181.33) | 339.26(254.73 to 447.85) | 2.94 (2.29 to 3.59) |
| Egypt | 83921.23(57448.86 to 109172.55) | 219679.72(133159.96 to 326113.2) | 284.84(195.21 to 370.15) | 345.52(212.84 to 505.58) | 0.84 (0.77 to 0.91) |
| El Salvador | 6396.25(5229.96 to 7628.79) | 37490.48(27567.39 to 50308.87) | 201.81(164.5 to 241.7) | 639.93(468.41 to 856.6) | 4.34 (3.63 to 5.04) |
| Equatorial Guinea | 464.12(304.79 to 632.23) | 1165.51(777.99 to 1731.8) | 216.06(146.53 to 293.91) | 219.84(150.49 to 319.14) | 0.22 (0.06 to 0.39) |
| Eritrea | 2237.61(1474.63 to 3322.9) | 6131.24(4168.53 to 8845.79) | 194.21(128.37 to 283.23) | 206.04(140.98 to 289.62) | 0.12 (-0.01 to 0.26) |
| Estonia | 456.28(353.39 to 569.58) | 1635.76(1198.27 to 2159.44) | 22.89(17.85 to 28.71) | 63.17(46.92 to 82.58) | 3.62 (3.13 to 4.12) |
| Eswatini | 870.92(621.25 to 1173.38) | 2634.46(1753.18 to 3746.09) | 268.08(193.22 to 352.86) | 409.36(279.43 to 569.17) | 1.7 (1.02 to 2.38) |
| Ethiopia | 69742.25(52791.94 to 88286.51) | 78902.68(63759.83 to 95006.39) | 310.92(239.77 to 388.98) | 179.17(144.73 to 214.88) | -1.97 (-2.11 to -1.83) |
| Fiji | 2005.93(1569.53 to 2518.61) | 4419.76(3447.56 to 5559.16) | 435.54(346.79 to 543.28) | 548.77(431.97 to 685.43) | 0.33 (-0.01 to 0.67) |
| Finland | 1917.79(1546.73 to 2360.09) | 3802.82(3021.61 to 4736.03) | 27.43(22.26 to 33.55) | 31.25(25.11 to 38.37) | 1.26 (0.89 to 1.64) |
| France | 28349.76(21737.21 to 35550.63) | 43580.88(33293.14 to 56514.21) | 33.97(26.35 to 42.53) | 31.34(24.33 to 39.67) | -0.34 (-0.45 to -0.24) |
| Gabon | 1518.43(1042.69 to 2047.21) | 3109.51(1880.17 to 4315.34) | 262.97(180.23 to 356.27) | 286.19(174.56 to 392.47) | 0.24 (0 to 0.48) |
| Gambia | 816.04(570.87 to 1131.31) | 2437.82(1768.1 to 3255.57) | 207.88(147.47 to 285.75) | 230.09(171.36 to 306.17) | 0.29 (0.14 to 0.45) |
| Georgia | 3765.33(2904.78 to 4673.86) | 6385.78(4926.16 to 7975.72) | 61.6(48.17 to 76.13) | 116.75(90.22 to 146.18) | 3.51 (2.83 to 4.19) |
| Germany | 66248.75(52093.73 to 82331.85) | 142168.24(109662.59 to 183642.03) | 52.46(41.57 to 64.29) | 68.54(53.9 to 87.09) | 1.32 (1.14 to 1.5) |
| Ghana | 15049.27(10611.67 to 21274.97) | 45777.16(31310.93 to 62427.63) | 212.1(151.26 to 291.96) | 254.21(177.38 to 340.38) | 0.88 (0.71 to 1.05) |
| Greece | 14057.79(10681.58 to 18047.55) | 17208.67(13023.02 to 22336.35) | 92.79(71.13 to 118) | 70.12(54.85 to 87.37) | -0.69 (-1.02 to -0.35) |
| Greenland | 27.27(21.01 to 34.28) | 44.83(32.61 to 59.12) | 81.42(63.58 to 101.98) | 67.07(49.69 to 88.08) | -0.79 (-0.94 to -0.65) |
| Grenada | 212.43(176.24 to 250.01) | 485.75(397.59 to 583.24) | 313.02(258.79 to 369.85) | 426.1(351.83 to 508.69) | 1.48 (1.33 to 1.63) |
| Guam | 222.35(187.03 to 259.12) | 719.46(589.09 to 875.51) | 253.29(217.01 to 294.55) | 384.12(315.45 to 466.05) | 1.74 (1.57 to 1.91) |
| Guatemala | 10389.87(8428.36 to 12432.73) | 58391.65(44325.32 to 75091.89) | 257.92(209.9 to 309.11) | 500.01(378.52 to 640.7) | 3.09 (2.67 to 3.52) |
| Guinea | 8583.57(6052.14 to 11724.81) | 15085.23(10888.56 to 20123.17) | 246.37(174.28 to 334.7) | 247.84(180.84 to 328.97) | 0.01 (-0.09 to 0.11) |
| Guinea-Bissau | 1856.81(1325.29 to 2534.34) | 2922.88(2097.65 to 3882.81) | 402.89(295.21 to 540.63) | 333.77(243.28 to 439.66) | -0.56 (-0.63 to -0.5) |
| Guyana | 1156.68(932.36 to 1401.8) | 3093.29(2284.66 to 4039.65) | 267.01(215.2 to 323.43) | 452.48(337.3 to 587.58) | 2.51 (2.28 to 2.73) |
| Haiti | 11558.22(8468.56 to 16521.98) | 24278.85(16548.1 to 37837.03) | 310.59(226.12 to 444.87) | 303.2(206.76 to 474.06) | 0.29 (0.13 to 0.45) |
| Honduras | 5494.39(4124.95 to 7442.67) | 30521.97(22779.07 to 41021) | 237.03(174.78 to 327) | 482.42(361.07 to 644.44) | 2.83 (2.57 to 3.08) |
| Hungary | 6949.48(5499.21 to 8451.56) | 11089.04(8564.04 to 14174.61) | 49.26(39.11 to 59.79) | 60.04(46.81 to 76.37) | 1.41 (1.13 to 1.68) |
| Iceland | 59.3(46.03 to 75.15) | 121.47(93.62 to 159.32) | 20.83(16.1 to 26.21) | 22.03(16.97 to 28.79) | 0.17 (-0.28 to 0.64) |
| India | 851917.47(645590.22 to 1068531.01) | 2236846.06(1724735.14 to 2801598.62) | 170.62(130.57 to 212.83) | 186.65(145.52 to 232.81) | 0.36 (0.12 to 0.6) |
| Indonesia | 371855.89(313174.38 to 435601.11) | 681464.06(554125.64 to 832217.07) | 283.88(238.55 to 327.93) | 272.71(224.02 to 328.98) | -0.12 (-0.21 to -0.03) |
| Iran (Islamic Republic of) | 47894.82(39353.14 to 56000.85) | 108024.44(91215.36 to 125075.56) | 179.44(149.06 to 209.35) | 147.63(123.85 to 171.36) | -0.84 (-0.95 to -0.72) |
| Iraq | 31620.26(24360.77 to 41566.93) | 81684.52(60910.42 to 107903.61) | 388.88(297.15 to 512.07) | 345.85(264.22 to 446.88) | -0.51 (-0.55 to -0.47) |
| Ireland | 2236.03(1702.17 to 2848.41) | 3156.03(2450.65 to 4027.28) | 54.9(42.41 to 68.92) | 42.43(33.09 to 53.8) | -1.28 (-1.47 to -1.09) |
| Israel | 5478.88(4235.09 to 6862.77) | 13736.65(10654.89 to 16982.06) | 114.29(89.34 to 142.78) | 116.84(90.94 to 144.13) | 1.1 (0.26 to 1.95) |
| Italy | 41163.28(31889.75 to 51211.94) | 53362.48(41493.47 to 67581.34) | 47.01(36.68 to 57.95) | 35.77(28.06 to 44.81) | -1.03 (-1.32 to -0.74) |
| Jamaica | 3346.72(2812.03 to 3880.79) | 8819.84(6811.1 to 11224.89) | 189.16(157.83 to 219.82) | 294.7(228.06 to 375.78) | 1.06 (0.57 to 1.56) |
| Japan | 190775.8(161431.27 to 219904.37) | 286796.8(235237.78 to 337376.57) | 114.88(97.51 to 132.17) | 84.23(69.86 to 99.78) | -0.9 (-1.13 to -0.67) |
| Jordan | 4488.32(3604.02 to 5458.9) | 19109.18(15126.31 to 23397.72) | 328.02(261.68 to 402.04) | 291.06(231.03 to 357.38) | -0.36 (-0.55 to -0.17) |
| Kazakhstan | 10369.58(8193.36 to 12770.94) | 19472.29(15077.49 to 24007.39) | 74.45(59.27 to 90.56) | 107.79(84.28 to 132.11) | 0.67 (0.34 to 1) |
| Kenya | 11198.11(8684.53 to 14422.02) | 37581.54(28979.32 to 47616.88) | 128.87(98.93 to 167.35) | 160.78(123.7 to 201.29) | 0.87 (0.75 to 1) |
| Kiribati | 298.87(235.5 to 367.76) | 675.11(486.46 to 896.59) | 631.58(503.49 to 771.31) | 774.35(572.52 to 1027.54) | 0.42 (0.02 to 0.81) |
| Kuwait | 1471.46(1210.39 to 1729.48) | 3486.03(2796.76 to 4325.12) | 215.59(175.09 to 254.18) | 130.73(103.63 to 161.47) | -1.99 (-2.66 to -1.32) |
| Kyrgyzstan | 4129.63(3133.82 to 5294.98) | 5335.45(4146.24 to 6851.44) | 124.26(95.01 to 158.63) | 97.9(76.59 to 124.79) | -1.44 (-1.85 to -1.04) |
| Lao People's Democratic Republic | 17594.42(13327.73 to 22843.53) | 28120.38(20878.57 to 37327.59) | 708.16(533.92 to 913.18) | 541.63(409.73 to 711.01) | -1.08 (-1.16 to -1.01) |
| Latvia | 537.86(410.52 to 691.2) | 1262.22(953.95 to 1639.38) | 15.53(11.94 to 19.98) | 35.26(26.9 to 45.99) | 3.55 (3.2 to 3.91) |
| Lebanon | 5488.18(4248.48 to 6996.48) | 9220.6(6792.16 to 12269.13) | 241.9(189.87 to 306.94) | 176.74(129.31 to 235.27) | -1.01 (-1.21 to -0.82) |
| Lesotho | 1647.58(1162.52 to 2276.48) | 5043.12(3286.6 to 7090.4) | 160.54(113.09 to 220.44) | 369.43(247.47 to 508.44) | 3.58 (3.22 to 3.94) |
| Liberia | 3323.64(2450.89 to 4357.43) | 5751.46(3842.52 to 8298.79) | 288.36(215.2 to 376.49) | 240.7(165.11 to 341.15) | -0.39 (-0.7 to -0.09) |
| Libya | 4479.41(3262.46 to 5882.29) | 13147.76(8938.77 to 17914.92) | 231.79(168.71 to 306.97) | 247.93(168.35 to 336.56) | 0.39 (0.25 to 0.53) |
| Lithuania | 809.17(619.99 to 1020.81) | 1438.55(1095.07 to 1813.31) | 18.33(14.12 to 23.04) | 28.33(21.64 to 36.35) | 1.15 (0.7 to 1.6) |
| Luxembourg | 273.49(210.81 to 346.84) | 491.49(377.3 to 640.99) | 50.62(39.61 to 63.69) | 48.1(37.18 to 62.61) | 0.02 (-0.19 to 0.22) |
| Madagascar | 8828.86(6533.3 to 11895.3) | 17264.23(12323.44 to 23939.89) | 157.63(116.84 to 211.66) | 144.18(103.13 to 196.69) | -0.38 (-0.45 to -0.31) |
| Malawi | 7662.02(5802.63 to 9663.82) | 14065.62(10540.03 to 18069.94) | 186.33(143.2 to 231.09) | 182.08(138.93 to 229.88) | -0.25 (-0.44 to -0.07) |
| Malaysia | 34344.58(29773.06 to 38887.12) | 86982.62(68679.82 to 108870.73) | 319.55(275.47 to 361.51) | 308.15(244.13 to 382.1) | -0.53 (-0.7 to -0.35) |
| Maldives | 693.52(569.04 to 854.62) | 1153.14(948.48 to 1369.39) | 657.4(548.97 to 801.59) | 331.72(268.25 to 398.74) | -2.75 (-3.06 to -2.44) |
| Mali | 11778.06(8447.08 to 15836.14) | 21650.52(15711.81 to 29725.3) | 263.27(193.28 to 345.55) | 226.72(166.65 to 303.06) | -0.36 (-0.54 to -0.18) |
| Malta | 283.19(216.17 to 360.18) | 492.45(372.58 to 635.09) | 67.34(51.77 to 85.69) | 53.98(41.64 to 68.91) | -0.69 (-0.87 to -0.51) |
| Marshall Islands | 104.03(81.99 to 134.25) | 292.86(208.15 to 405.84) | 494.89(390.15 to 644.52) | 673.26(483.37 to 930.91) | 1.01 (0.74 to 1.27) |
| Mauritania | 3710.48(2763.32 to 4780.17) | 4834.92(3352.01 to 6618.09) | 348.36(259.9 to 447.47) | 219.95(155.16 to 297.61) | -1.47 (-1.59 to -1.36) |
| Mauritius | 4936.78(4339.49 to 5483.92) | 17847.16(14168.94 to 22193.03) | 599.48(526.07 to 666.47) | 1023.49(813.85 to 1268.02) | 2.21 (1.83 to 2.59) |
| Mexico | 111427.57(94973.81 to 127839.56) | 645928.97(515349.2 to 792846.29) | 238.89(202.13 to 273.48) | 533.54(425.04 to 649.67) | 3.07 (2.62 to 3.52) |
| Micronesia (Federated States of) | 364.61(280.19 to 471.33) | 804.47(524.61 to 1113.03) | 641.76(495.81 to 818.9) | 972.55(659.5 to 1321.21) | 1.34 (0.97 to 1.72) |
| Monaco | 19.21(14.58 to 25.12) | 32.51(24.41 to 42.51) | 28.16(21.58 to 36.33) | 34.42(26.05 to 44.25) | 0.92 (0.68 to 1.17) |
| Mongolia | 3084.86(2300.17 to 4038.06) | 4226.58(3022.47 to 5712.22) | 264(194.93 to 342.78) | 149.27(108.92 to 197.85) | -3.12 (-3.55 to -2.68) |
| Montenegro | 539.67(413.45 to 680.95) | 920.84(687.82 to 1183.22) | 85.85(66.07 to 107.71) | 96.22(72.91 to 123.43) | 0.37 (0.23 to 0.52) |
| Morocco | 29452.19(21961.98 to 38120.55) | 81890.25(59860.09 to 107619.08) | 210.08(157.83 to 278.02) | 262.79(194.18 to 342.67) | 1.12 (0.99 to 1.25) |
| Mozambique | 9469.8(6855.29 to 12843.87) | 21457.28(15474.83 to 28728.97) | 151.43(110.02 to 203.27) | 180.32(131.91 to 239.43) | 0.68 (0.56 to 0.79) |
| Myanmar | 133743.64(100470.77 to 175277.77) | 188667.21(147998.08 to 240841.82) | 457.21(348.48 to 587.14) | 368.11(292.3 to 462.89) | -0.85 (-0.98 to -0.72) |
| Namibia | 1263.04(829.94 to 1906.54) | 2473.83(1670.71 to 3647.11) | 169.92(112.48 to 252.72) | 168.34(114.32 to 247.45) | -0.45 (-0.89 to -0.02) |
| Nauru | 34.7(24.84 to 44.74) | 53.15(35.28 to 70.55) | 626.41(460.57 to 800.17) | 837.89(591.41 to 1093.74) | 0.89 (0.55 to 1.23) |
| Nepal | 15303.58(10216.37 to 21439.59) | 48870.02(34101.64 to 66890.13) | 145.28(97.81 to 201.56) | 210.42(148.4 to 287.63) | 1.38 (1.07 to 1.69) |
| Netherlands | 6815.05(5252.32 to 8648.72) | 12730.23(9797.88 to 16398.29) | 34.17(26.35 to 43.28) | 37.1(28.68 to 47.22) | 0.38 (0.21 to 0.54) |
| New Zealand | 1284.81(993.53 to 1629.77) | 3147.6(2430.99 to 3989.24) | 33.84(26.05 to 42.89) | 42.56(32.9 to 54.37) | 0.94 (0.62 to 1.26) |
| Nicaragua | 5774.27(4672.85 to 6869.11) | 33207.97(25251.34 to 42562.68) | 337.2(271.76 to 402.61) | 706.11(540.32 to 897.54) | 3 (2.71 to 3.29) |
| Niger | 7719.01(5519.58 to 10411.06) | 17731.8(12535.23 to 24503.84) | 242.22(174.69 to 329.24) | 203.12(146.14 to 272.12) | -0.53 (-0.64 to -0.43) |
| Nigeria | 75831.51(53753.3 to 104953.17) | 144062.26(104158.29 to 193970.05) | 166.15(119.45 to 230.14) | 152.6(111.88 to 201.67) | -0.52 (-0.71 to -0.33) |
| Niue | 9.3(7.16 to 11.82) | 11.69(8.22 to 14.95) | 443.08(336.7 to 564.55) | 565.02(397.01 to 732.4) | 0.66 (0.41 to 0.91) |
| North Macedonia | 1287.45(955.02 to 1677.74) | 2546.78(1850.1 to 3348.04) | 66.31(49.28 to 85.54) | 79.6(58.88 to 104.23) | 0.37 (0.07 to 0.67) |
| Northern Mariana Islands | 141.78(107.47 to 184.63) | 338.25(268.16 to 420.01) | 517.29(418.5 to 650.47) | 617.85(498.93 to 757.5) | 0.75 (0.66 to 0.84) |
| Norway | 1813.03(1392.56 to 2324.19) | 2957.13(2313.86 to 3752.19) | 27.04(20.81 to 34.08) | 30.42(23.94 to 38.17) | 0.56 (0.41 to 0.7) |
| Oman | 901.68(666.37 to 1196.25) | 2616.51(2108.41 to 3183.71) | 135.39(101.31 to 178.84) | 157.27(128.06 to 188.21) | 0.78 (0.64 to 0.93) |
| Pakistan | 120867.99(86307.68 to 168574.05) | 417761.84(294726.2 to 561551.63) | 197.93(141.47 to 276.18) | 324.3(230.09 to 436.65) | 1.71 (1.43 to 1.99) |
| Palau | 77.12(59.38 to 100.93) | 196.08(147.03 to 254.87) | 674.16(531.07 to 880.08) | 867(659.79 to 1107.73) | 0.83 (0.64 to 1.03) |
| Palestine | 3136.05(2319.41 to 4121.67) | 6535.63(5203.56 to 8028.97) | 353.04(263.72 to 464.35) | 268.98(216.52 to 326.67) | -0.67 (-0.92 to -0.42) |
| Panama | 2152.37(1771.23 to 2537.85) | 9988.13(7600.7 to 12855.86) | 135.94(111.22 to 159.92) | 240.01(182.57 to 308.99) | 1.99 (1.67 to 2.3) |
| Papua New Guinea | 4494.13(3497.14 to 5599.13) | 13731.84(10539.25 to 17844.37) | 176.26(139.03 to 218.53) | 205.23(157.57 to 262.82) | 0.45 (0.32 to 0.58) |
| Paraguay | 2982.78(2459.87 to 3511.71) | 16779.8(12384.27 to 21933.27) | 127.76(105.78 to 150.59) | 293.74(218.08 to 381.67) | 3.46 (3.24 to 3.68) |
| Peru | 20788.69(16051.8 to 25492.93) | 54345.41(40107.48 to 73177.69) | 163.78(127.9 to 201.29) | 168.04(124.1 to 225.98) | 0.18 (0 to 0.36) |
| Philippines | 149908.38(127606.71 to 173336.55) | 504695.54(400115.91 to 619582.3) | 411.76(352.58 to 475.36) | 560.67(452.72 to 682.94) | 1.54 (1.26 to 1.82) |
| Poland | 33212.25(26203.73 to 40294.96) | 30920.28(24255.55 to 38609.34) | 76.57(60.87 to 92.42) | 46.23(36.41 to 57.67) | -1.43 (-1.83 to -1.02) |
| Portugal | 8795.36(6617.19 to 11187.24) | 14845.19(11293.97 to 19282.09) | 64.83(49.34 to 81.38) | 59.37(45.79 to 75.54) | -0.09 (-0.59 to 0.42) |
| Puerto Rico | 8613.23(7215.13 to 9877.61) | 15698.45(12126.92 to 19907.02) | 239.23(201.04 to 274.08) | 246.35(190.54 to 310.92) | 0.58 (0.36 to 0.81) |
| Qatar | 329.02(253.4 to 455.39) | 1936.92(1495.83 to 2484.53) | 331.98(250.61 to 519.76) | 276.23(214.92 to 354.37) | -0.43 (-0.73 to -0.13) |
| Republic of Korea | 47053.92(43302.47 to 50930.3) | 87806.07(77973.94 to 97996.39) | 144.08(131.98 to 157.68) | 100.02(89.1 to 111.73) | -1.09 (-1.2 to -0.98) |
| Republic of Moldova | 827.61(644.09 to 1039.95) | 1793.85(1349.64 to 2305.19) | 19.09(14.95 to 23.64) | 32.58(24.7 to 41.79) | 1.8 (1.47 to 2.13) |
| Romania | 9937.82(6567.24 to 12758.9) | 17025.18(12738.54 to 22093.45) | 36(24 to 46.18) | 49.02(36.72 to 62.8) | 1.67 (1.29 to 2.05) |
| Russian Federation | 75668.19(59410.17 to 95329.45) | 88837.3(68752.19 to 110980.36) | 42.92(33.87 to 54.03) | 40.33(31.5 to 50.31) | -0.69 (-0.96 to -0.42) |
| Rwanda | 7704(5707.58 to 9821.51) | 10622.12(7711.77 to 13885.73) | 245.17(186.14 to 312.41) | 169.75(127.16 to 218.85) | -2.12 (-2.48 to -1.77) |
| Saint Kitts and Nevis | 146.82(120.54 to 173.44) | 309.59(227.94 to 394.17) | 420.37(349.95 to 495.9) | 445.03(337.48 to 557.86) | 0.3 (0.14 to 0.46) |
| Saint Lucia | 247.27(204.54 to 288.83) | 654.79(518.98 to 791.99) | 277.23(229.67 to 323.4) | 302.88(241.26 to 365.16) | 0.45 (0.27 to 0.64) |
| Saint Vincent and the Grenadines | 168.07(139.61 to 195.23) | 448.58(362.77 to 537.93) | 230.61(191.4 to 268.27) | 334.84(272.22 to 399.73) | 1.47 (1.32 to 1.61) |
| Samoa | 440.38(322.55 to 579.95) | 865.24(656.73 to 1125.91) | 454.14(338.79 to 588.97) | 541.89(416.59 to 696.26) | 0.52 (0.31 to 0.73) |
| San Marino | 8.2(6.3 to 10.81) | 18.43(13.2 to 25.64) | 25.4(19.58 to 33.43) | 28.38(20.5 to 38.75) | 0.75 (0.62 to 0.88) |
| Sao Tome and Principe | 187.1(135.22 to 239.02) | 403.6(295.04 to 534.98) | 284.94(209.19 to 362.92) | 345.85(255.31 to 452.03) | 0.5 (0.3 to 0.7) |
| Saudi Arabia | 23980.4(17259.63 to 31984.64) | 82320.48(61154.26 to 108334.48) | 379.54(275.05 to 511.7) | 406.93(307.82 to 528.78) | 0.41 (0.17 to 0.66) |
| Senegal | 10074.52(7188.29 to 13579.38) | 20541.23(14498.02 to 28191.92) | 289.28(208.36 to 392.87) | 254.24(182.2 to 343.46) | -0.33 (-0.5 to -0.16) |
| Serbia | 10253.86(7437.41 to 13492.19) | 14771.6(10569.92 to 19669.92) | 90.51(67.53 to 117.22) | 93.74(68.03 to 124.08) | -0.36 (-0.53 to -0.19) |
| Seychelles | 211.86(180.71 to 242.62) | 578.35(491.13 to 671.72) | 369.06(314.67 to 421.89) | 508.63(434.22 to 586.56) | 0.64 (0.31 to 0.97) |
| Sierra Leone | 4246.64(3110.75 to 5637.85) | 8399.56(6074.07 to 11339.4) | 210.08(154.49 to 277.49) | 205.24(150.27 to 273.68) | 0.15 (0.04 to 0.27) |
| Singapore | 4490.14(3921.31 to 5025.43) | 9212.69(7798 to 10679.54) | 189.52(165.77 to 212.39) | 117.85(99.85 to 136.54) | -0.19 (-0.63 to 0.25) |
| Slovakia | 5793.6(4391.54 to 7181.2) | 5804.91(4326.7 to 7629.83) | 97.74(74.26 to 120.34) | 64.73(48.54 to 84.49) | -0.45 (-0.88 to -0.01) |
| Slovenia | 829.91(643.26 to 1059.09) | 1280.24(955.76 to 1706.86) | 34.3(26.45 to 43.7) | 30.76(23.01 to 40.71) | -0.19 (-0.35 to -0.03) |
| Solomon Islands | 1326.8(966.79 to 1792.37) | 1940.43(1490.92 to 2455.85) | 676.63(489.97 to 909.2) | 421.52(332.47 to 523.86) | -1.8 (-2.18 to -1.41) |
| Somalia | 6722.69(4676.92 to 9320.86) | 16593.39(11763.24 to 22930.13) | 235.74(164.81 to 322.75) | 222.48(158.98 to 305.33) | -0.07 (-0.11 to -0.03) |
| South Africa | 34207.46(26304.52 to 43034.72) | 93110.65(73169.47 to 114201.4) | 144.99(113.1 to 180.69) | 200.76(158.51 to 246.21) | 1.8 (1.49 to 2.12) |
| South Sudan | 4686.95(3237.66 to 6589.04) | 7294.45(4976.23 to 10402.02) | 188.03(130.81 to 268.21) | 183.02(126.64 to 253.3) | -0.11 (-0.15 to -0.07) |
| Spain | 32035.19(23875.27 to 40576.79) | 37809.91(28601.56 to 49888.23) | 59.42(44.99 to 74.32) | 36.11(27.73 to 46.48) | -1.76 (-1.9 to -1.61) |
| Sri Lanka | 38456.68(32616.51 to 44112.87) | 76087.1(57449.94 to 100368.82) | 306.61(262.11 to 351.91) | 301.44(228.26 to 395.77) | 0.08 (-0.19 to 0.35) |
| Sudan | 19362.81(13760.4 to 26972.73) | 40217.11(27172.9 to 59595.85) | 198.34(139.61 to 280.75) | 207.04(140.05 to 311.27) | -0.13 (-0.36 to 0.1) |
| Suriname | 699.5(565.29 to 828.63) | 2471.2(1956.38 to 3022.85) | 251.87(205.63 to 298.24) | 402.23(320.46 to 489.06) | 1.58 (1.32 to 1.84) |
| Sweden | 4530.35(3669.65 to 5546.9) | 8853.61(7165.51 to 10750.38) | 30.48(24.74 to 37.34) | 40.43(32.91 to 48.97) | 1.1 (1.02 to 1.18) |
| Switzerland | 3817.99(3004.66 to 4767.83) | 8053.53(6296.81 to 10282.83) | 36.54(29.05 to 44.91) | 42.99(33.65 to 54.19) | 0.67 (0.6 to 0.74) |
| Syrian Arab Republic | 15595.64(11680.88 to 19983.21) | 24759.27(18151.48 to 33267.52) | 273.08(204.73 to 353.9) | 200.84(149.99 to 264.34) | -1.65 (-1.95 to -1.35) |
| Taiwan (Province of China) | 43521.58(38240.73 to 48586.4) | 92723.73(73960.99 to 114289.88) | 268.75(236.54 to 299.06) | 238.98(190.58 to 293.7) | -0.36 (-0.51 to -0.21) |
| Tajikistan | 1108.53(892.19 to 1372.73) | 4539.24(3470.44 to 5896.77) | 34.58(27.76 to 42.32) | 78.17(60.28 to 99.61) | 3.02 (2.69 to 3.36) |
| Thailand | 164323.79(137907.52 to 196423.93) | 322753.36(240210.08 to 419442.98) | 375.75(320.72 to 439.52) | 325.35(243.73 to 421.01) | -0.63 (-0.81 to -0.45) |
| Timor-Leste | 1564.6(1131.35 to 2154.81) | 3285.95(2290.9 to 4347.54) | 399.08(297.15 to 548.81) | 375.98(268.88 to 492.53) | -0.26 (-0.51 to 0) |
| Togo | 3395.72(2443.75 to 4587.44) | 9328.96(6580.56 to 12570.39) | 237.3(174.15 to 316.5) | 223.48(163.52 to 296.6) | -0.17 (-0.22 to -0.11) |
| Tokelau | 5.44(4.03 to 7.32) | 6.64(4.9 to 9.18) | 420.14(311.02 to 570.62) | 499.28(368.93 to 689.49) | 0.57 (0.36 to 0.78) |
| Tonga | 212.6(166.55 to 284.37) | 407.97(309.47 to 557.12) | 351.88(278.01 to 468.95) | 496.73(376.9 to 672.39) | 1.14 (0.81 to 1.47) |
| Trinidad and Tobago | 1626.07(1339.52 to 1908.29) | 5502.26(3974.22 to 7358.01) | 184.31(152.23 to 216.24) | 298.03(215.4 to 398.26) | 1.92 (1.66 to 2.17) |
| Tunisia | 7822.32(6014.6 to 9913.38) | 20173.68(14575.63 to 27154.43) | 154.6(119.45 to 196.11) | 161.55(116.64 to 216.27) | 0.2 (0.12 to 0.28) |
| Turkey | 101428.29(78427.5 to 136460.95) | 160102.22(124680.35 to 201880.19) | 276.92(214.3 to 377.97) | 182.73(142.06 to 229.82) | -0.96 (-1.17 to -0.76) |
| Turkmenistan | 2955.13(2317.07 to 3634.84) | 8228.57(6046.74 to 10845.5) | 131.33(103.06 to 159.85) | 182.5(136.03 to 237.28) | 0.66 (0.38 to 0.95) |
| Tuvalu | 37.95(28.75 to 49.29) | 65.3(47.02 to 90.38) | 504.5(384.93 to 653.91) | 609.45(441.81 to 835.02) | 0.66 (0.48 to 0.85) |
| Uganda | 11895.27(8737.16 to 16099.12) | 27203.17(19677.23 to 36069.22) | 177.13(129.82 to 236.32) | 181.31(134.1 to 240.33) | -0.22 (-0.38 to -0.05) |
| Ukraine | 10483.67(8228.52 to 13335.03) | 18041.54(13671.02 to 23453.96) | 15.49(12.21 to 19.65) | 26.69(20.46 to 34.82) | 2.43 (2.1 to 2.77) |
| United Arab Emirates | 2711.7(1987.48 to 3658.38) | 23685.72(15112.33 to 37954.07) | 478.89(324.58 to 624.21) | 400.98(266.17 to 625.26) | -0.58 (-1.02 to -0.14) |
| United Kingdom | 26868.76(20758.12 to 33608) | 34503.62(26905.63 to 43203.22) | 30.53(23.89 to 38.08) | 28.17(21.98 to 35.32) | -0.03 (-0.18 to 0.12) |
| United Republic of Tanzania | 17300.1(12751.52 to 23000.34) | 39079.67(31040.9 to 48372.2) | 152.75(113.57 to 199.89) | 156.29(124.37 to 192.28) | 0.06 (-0.01 to 0.12) |
| United States of America | 203821.19(160637.74 to 249054.93) | 760939.89(617376.14 to 905048.55) | 65.68(51.63 to 80.74) | 140.99(115.1 to 166.77) | 2.77 (2.43 to 3.11) |
| United States Virgin Islands | 177.46(142 to 216.25) | 458.59(362.17 to 558.69) | 192.44(154.55 to 233.11) | 264.53(211.3 to 321.82) | 1.55 (1.36 to 1.75) |
| Uruguay | 3352.97(3084.29 to 3672.55) | 5866.51(5065.58 to 6760.94) | 87.38(80.32 to 95.53) | 111.76(97.44 to 127.43) | 1.29 (1.06 to 1.52) |
| Uzbekistan | 18345.59(13739.37 to 25273.93) | 48213.48(36972.9 to 61085.4) | 144.36(106.07 to 202.63) | 189.93(148.7 to 234.39) | 0.52 (-0.18 to 1.22) |
| Vanuatu | 256.24(174.29 to 362.74) | 1078.93(760.31 to 1482.55) | 315.46(219.62 to 444) | 535.46(381.67 to 734.75) | 1.89 (1.75 to 2.02) |
| Venezuela (Bolivarian Republic of) | 17239.56(14205.26 to 20040.18) | 101190.39(74537.05 to 135807.9) | 161.97(133.97 to 188.02) | 339.12(250.39 to 451.35) | 2.09 (1.63 to 2.55) |
| Viet Nam | 141932.21(109441.34 to 181334.84) | 274152.9(207779.58 to 353140.35) | 325.24(254.06 to 411.06) | 284.1(218.08 to 360.34) | -0.47 (-0.92 to -0.01) |
| Yemen | 10177.97(7007.59 to 14874.95) | 26255.06(18708.87 to 35727.84) | 193.94(133.64 to 282.24) | 184.9(133.52 to 253.53) | -0.21 (-0.3 to -0.12) |
| Zambia | 7217.42(5418.92 to 9214.15) | 17292.64(12719.13 to 22783.9) | 230.81(173.24 to 292.37) | 230.08(172.09 to 302.42) | -0.32 (-0.55 to -0.09) |
| Zimbabwe | 6063.81(4136.13 to 9737.56) | 18098.92(11941.16 to 27905.24) | 140.63(96.76 to 221.51) | 236.84(157.95 to 362.46) | 2.09 (1.72 to 2.47) |

ASDR, age-standard DALYs rate; DALYs, disability-adjusted life years; EAPC, estimated annual percentage change.
